# The action of physiological and synthetic steroids on the calcium channel CatSper in human sperm

**DOI:** 10.3389/fcell.2023.1221578

**Published:** 2023-07-20

**Authors:** Lydia Wehrli, Ioannis Galdadas, Lionel Voirol, Martin Smieško, Yves Cambet, Vincent Jaquet, Stéphane Guerrier, Francesco Luigi Gervasio, Serge Nef, Rita Rahban

**Affiliations:** ^1^ Department of Genetic Medicine and Development, University of Geneva, Geneva, Switzerland; ^2^ Swiss Centre for Applied Human Toxicology (SCAHT), Basel, Switzerland; ^3^ Institute of Pharmaceutical Sciences of Western Switzerland, University of Geneva, Geneva, Switzerland; ^4^ School of Pharmaceutical Sciences, University of Geneva, Geneva, Switzerland; ^5^ Research Center for Statistics, Geneva School of Economics and Management, University of Geneva, Geneva, Switzerland; ^6^ Department of Pharmaceutical Sciences, University of Basel, Basel, Switzerland; ^7^ Readers, Assay Development and Screening Unit (READS Unit), Faculty of Medicine, University of Geneva, Geneva, Switzerland; ^8^ Faculty of Science, University of Geneva, Geneva, Switzerland; ^9^ Department of Chemistry, University College London, London, United Kingdom; ^10^ Institute of Structural and Molecular Biology, University College London, London, United Kingdom

**Keywords:** CatSper, steroids, human sperm, high-throughput screening, calcium signaling, pharmacophore modelling, acrosome reaction

## Abstract

The sperm-specific channel CatSper (cation channel of sperm) controls the intracellular Ca^2+^ concentration ([Ca^2+^]_i_) and plays an essential role in sperm function. It is mainly activated by the steroid progesterone (P4) but is also promiscuously activated by a wide range of synthetic and physiological compounds. These compounds include diverse steroids whose action on the channel is so far still controversial. To investigate the effect of these compounds on CatSper and sperm function, we developed a high-throughput screening (HTS) assay to measure changes in [Ca^2+^]_i_ in human sperm and screened 1,280 approved and off-patent drugs including 90 steroids from the Prestwick chemical library. More than half of the steroids tested (53%) induced an increase in [Ca^2+^]_i_ and reduced the P4-induced Ca^2+^ influx in human sperm in a dose-dependent manner. Ten of the most potent steroids (activating and P4-inhibiting) were selected for a detailed analysis of their action on CatSper and their ability to act on sperm acrosome reaction (AR) and penetration in viscous media. We found that these steroids show an inhibitory effect on P4 but not on prostaglandin E1-induced CatSper activation, suggesting that they compete for the same binding site as P4. Pregnenolone, dydrogesterone, epiandrosterone, nandrolone, and dehydroepiandrosterone acetate (DHEA) were found to activate CatSper at physiologically relevant concentrations within the nanomolar range. Like P4, most tested steroids did not significantly affect the AR while stanozolol and estropipate slightly increased sperm penetration into viscous medium. Furthermore, using a hybrid approach integrating pharmacophore analysis and statistical modelling, we were able to screen *in silico* for steroids that can activate the channel and define the physicochemical and structural properties required for a steroid to exhibit agonist activity against CatSper. Overall, our results indicate that not only physiological but also synthetic steroids can modulate the activity of CatSper with varying potency and if bound to CatSper prior to P4, could impair the timely CatSper activation necessary for proper fertilization to occur.

## Introduction

After being deposited in the female reproductive tract, spermatozoa undergo multiple complex processes before reaching and fertilizing the oocyte. Although motile upon ejaculation, sperm undergo a priming step referred to as capacitation which promotes their response to chemical cues (such as progesterone) and helps them locate the oocyte by chemotaxis. The sperm must then acquire a specific type of motility pattern characterized by an asymmetric flagellar beat movement known as hyperactivation, allowing them to break through the protective egg vestment and release digestive enzymes to penetrate the egg through the acrosome reaction (reviewed by Tosti and Menezo, 2016). These diverse processes are known to be partly controlled by the intracellular calcium concentration ([Ca^2+^]_i_) which is regulated almost exclusively by the principal sperm-specific Cationic channel of Sperm (CatSper) (reviewed by (Kaupp and Strünker, 2017; Rahban and Nef, 2020; Wang et al., 2021). Indeed, a suboptimal calcium influx is associated with reduced male fertility, and men with mutations in CATSPER are infertile (Avidan et al., 2003; Avenarius et al., 2009; Hildebrand et al., 2010; Smith et al., 2013; Williams et al., 2015; Brown et al., 2018; Luo et al., 2019; Schiffer et al., 2020).

CatSper is one of the most complex channels ever described (Zhao et al., 2022). Located specifically in the membrane of the flagellum, CatSper is a heterotetrameric channel composed of at least 10 subunits with four pore-forming α subunits (CatSper 1–4) and six auxiliary subunits, out of which four are transmembrane non-pore forming auxiliary subunits (β, γ, δ, and ε) and two are cytosolic (CatSper ζ and EFCAB9) (Quill et al., 2001; Carlson et al., 2003; Ren and Xia, 2010; Darszon et al., 2011; Chung et al., 2017; Hwang et al., 2019). Expression and association of the pore-forming and the transmembrane auxiliary CatSper subunits are required for channel function and fertilization. The cytosolic subunits EFCAB9 and the ζ are not required to form a functional channel but confer pH- and calcium-sensitivity to CatSper, respectively (Hwang et al., 2019). Two recent studies reported a novel C2 membrane-associated domain-containing protein encoded by *C3cd6* named CatSperT, which plays an important role in the spatiotemporal trafficking of the channel to the flagellar membrane (Hwang et al., 2022; Yang et al., 2022). Several previously uncharacterized transmembrane proteins in the CatSper structure, which have also been recently identified further complicate the mechanism of action, such as the testis-specific organic anion transporter SLCO6C1, located adjacent to CatSper 2 and ε, and three small transmembrane proteins (TMEM 262, TMEM 249, and an unassigned single transmembrane protein), which are located on the side of CatSper 4 and β (Zhao et al., 2022). How these proteins come together to orchestrate the function of CatSper and the physiological significance of the new CatSper subunits remain to be defined (Hwang and Chung, 2022). CatSper activation triggers a calcium influx and release from internal stores, resulting in oscillations in the [Ca^2+^]_i_ that propagate from the flagellum to the midpiece and head within seconds (Torrezan-Nitao et al., 2021). To enable rapid propagation of calcium signals along the flagellum, the CatSper channels form a complex of long zigzag lines resulting in four linear threads within the principal piece (Chung et al., 2014; Chung et al., 2017; Zhao et al., 2022).

CatSper is activated by alkalization of intracellular pH (pH_i_), membrane depolarization (Kirichok et al., 2006), and by natural ligands such as the steroid progesterone (P4) and the arachidonic acid derivative prostaglandins, mainly prostaglandin E1 (PGE1) (Lishko et al., 2011; Strünker et al., 2011). These ligands have distinct binding sites to activate the channel (Strünker et al., 2011), but the mechanism through which they activate CatSper is poorly understood and highly controversial. It has been suggested that P4 does not directly activate CatSper but rather binds to an upstream lipid hydrolase called the α/β hydrolase domain-containing protein 2 (ABHD2) (Miller et al., 2016). Upon P4 binding, ABHD2 is suggested to degrade the endocannabinoid 2-arachidonoylglycerol (2-AG), which blocks the channel and thereby releases CatSper from inhibition (Miller et al., 2016).

Studies over the last 30 years have shown that various endogenous and exogenous steroids are also able to evoke Ca^2+^ influx and motility responses in human sperm (Blackmore et al., 1990; Blackmore et al., 1996; Luconi et al., 1999; Luconi et al., 2004; Rossato et al., 2005; Brenker R. A. et al., 2018; Brenker S. C. et al., 2018; Rehfeld, 2020; Jeschke et al., 2021). Steroids such as 17-OH-progesterone, estradiol, dihydrotestosterone, androstenedione, and pregnenolone have been described to act via CatSper (Lishko et al., 2011; Strünker et al., 2011; Brenker R. A. et al., 2018; Brenker S. C. et al., 2018; Rehfeld, 2020; Jeschke et al., 2021). However, the activity of several steroids on CatSper is still a matter of controversy. For example, testosterone, estradiol, and hydrocortisone have been reported to be both CatSper antagonists (Mannowetz et al., 2017) and agonists (Brenker S. C. et al., 2018; Rehfeld, 2020). Furthermore, several studies demonstrated that CatSper is promiscuous and can be activated by a wide range of synthetic or natural compounds such as odorants (Spehr et al., 2003), endocannabinoids (Miller et al., 2016), and Endocrine Disrupting Chemicals (EDCs) (Tavares et al., 2013; Schiffer et al., 2014; Rehfeld et al., 2016). The list of compounds that can activate the channel was recently expanded to include commonly prescribed pharmaceutical drugs such as the selective serotonin reuptake inhibitors (SSRI) class of antidepressants (Rahban et al., 2021), the 5α reductase inhibitor finasteride (Birch et al., 2021), as well as the paracetamol metabolite N-arachidonoyl phenolamine (AM404) (Rehfeld et al., 2022). These results are consistent with the finding that human CatSper serves as a polymodal chemosensor that harbors promiscuous binding sites for structurally diverse ligands (Brenker et al., 2012). However, the physiological effects on sperm cells and the molecular characteristics that allow multiple structurally diverse steroids, whether physiological or synthetic, to activate CatSper remain poorly understood and contradictory.

Using our novel high-throughput screening (HTS) assay, we have developed an accurate and rapid method to investigate the effects of a wide range of pharmaceutical drugs, including synthetic and physiological steroids, on CatSper. We studied in detail 10 steroids that can directly activate the channel and interfere with the P4 but not the PGE1-induced Ca^2+^ response. We also evaluated the effect of these 10 steroids on the ligand-independent activation of the channel through intracellular alkalization and their effects on acrosome reaction and penetration into viscous media. Finally, pharmacophore and statistical modelling were used to screen *in silico* for steroids that can activate the channel and better understand the molecular characteristics of the steroids that promote CatSper activation.

## Materials and methods

### Reagents

The Prestwick library composed of 1,280 compounds was purchased by the R.E.A.D.S. unit of the University of Geneva. The drugs were dissolved at a stock concentration of 10 mM in dimethyl sulfoxide (DMSO). P4 and PGE1 were purchased from Sigma-Aldrich (Buchs, Switzerland). The fluorescent Ca^2+^ indicator Fluo-4 AM was purchased from Invitrogen and dissolved in DMSO at a final concentration of 2.5 µM (California, United States of America). The nuclear dye Propidium Iodide (PI) was purchased from Thermo Fisher Scientific (MA, United States of America), and diluted in water at a stock concentration of 1 mg/mL. Pisum sativum agglutinin (PSA) staining dye, was purchased from Sigma-Aldrich and dissolved in dissolved in Phosphate Buffered Saline (PBS) at a stock concentration of 1 mg/mL. Hoechst-33342 was purchased from Sigma-Aldrich (Buchs, Switzerland), and dissolved in water at a stock concentration of 1.5 mg/mL (Buchs, Switzerland). Human serum albumin (HSA) was obtained from Polygon Diagnostics (Lucerne, Switzerland). MDL 12330A hydrochloride was purchased from Tocris Bioscience (Bristol, UK). The following steroids were purchased from Sigma-Aldrich (Buchs, Switzerland): P4, dehydroisoandosterone 3-acetate (DHEA), estropipate, stanozolol, epiandrosterone, deoxycorticosterone, dydrogesterone, 3-alpha-Hydroxy-5-beta-androstan-17-one, pregnenolone, medrysone, equilin, estrone, norgestimate, canrenoic acid potassium salt, exemestane, finasteride, lithocholic acid, formestane, exemestane, fluorometholone, estradiol-17 beta, androsterone, medrysone, nandrolone, fluorometholone, oxymetholone, urosiol, oxandrolone, canrenone, fulvestrant, lynestrenol, norethindrone, ethisterone, nomegestrol acetate, gestrinone, chlormadinone acetate, norgestrel-(−)-D, ethynodiol diacetate, mifepristone, budesonide, adrenosterone, ethinylestradiol, megestrol acetate, ethynylestradiol 3-methyl ether, 3α,21-dihydroxy-5α-pregnan-20-one, 5α-pregnan-3α-ol-20-one, 19-norandrosterone, androsterone, 5alpha-pregnan-20-one, 5alpha-androstan-3-one, 5alpha-pregnane-3,20-dione, androstenedione, tanaproget, BB_NC-00182 and dissolved at a stock concentration of 20 mM in DMSO. To implement the pharmacophore model, the steroids were purchased from ChemDIV (California, United States) under the following ID numbers: N017–0003, N039–0025, N050–0010, 3647–1627, N044–0005, 0449–0076, 2361–0084, N039–0043, N050–0013, N039–0021, 0449–0077, N017–0007, 8011–0433, N050–0008, 3529–0011, 3529–0046, 5263–0009, N050-0018, and 3β,21-dihydroxy-5α-pregnan-20-one, 21-hydroxypregnenolone and MolPort-001-728-767 were purchased from MolPort (Riga, Latvia), and promegestrone was purchased from BOC Sciences (NY, United States) and dissolved at a 20 mM stock concentration in DMSO.

### Experimental design

**FIGURE 1:**
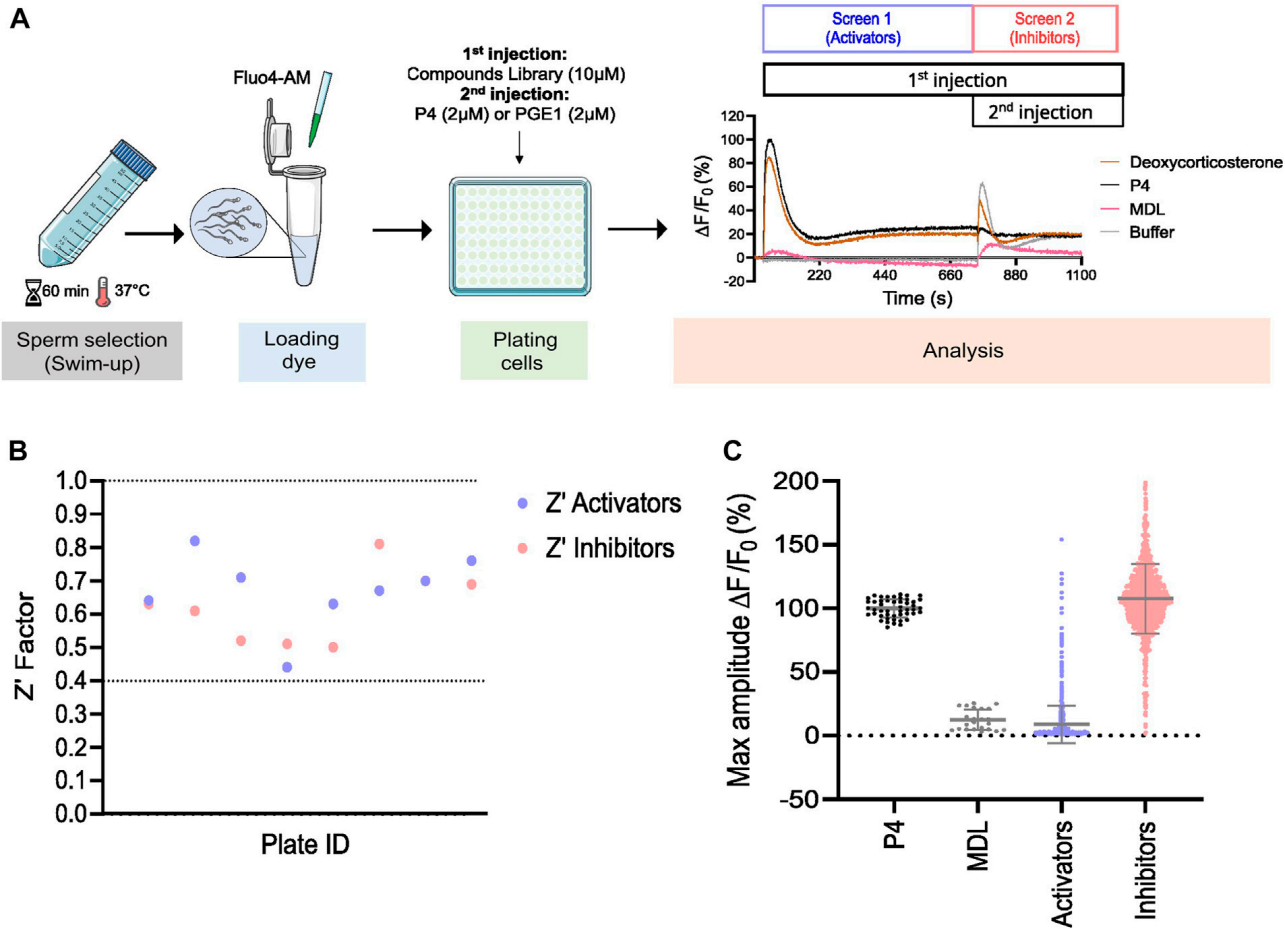
Prestwick library screen in human spermatozoa. **(A)** Graphical overview of the workflow used to screen the Prestwick library. Motile sperm were selected via ‘swim-up’ and labeled with the fluorescent dye Fluo4-AM to image changes in [Ca^2+^]_i_. Cells were then plated and the change in [Ca^2+^]_i_ was assessed simultaneously in all wells using a Functional Drug Screening System (FDSS/μCELL). Fluorescence was measured before and after the addition of compounds to evaluate whether they can induce an increase in [Ca^2+^]_i_ alone (Screen 1, activators). A second injection was then performed after 12 min to assess whether these compounds can alter the response of P4 and PGE1, the main ligands activating CatSper (Screen 2, inhibitors). Representative traces of Ca^2+^ influx by deoxycorticosterone (10 µM), P4 (2 µM), non-specific CatSper inhibitor MDL (at 20 µM) used as a negative control and DMSO, used as a vehicle control (buffer) are shown. **(B)** The standard high-throughput screening (HTS) metric, Z′ Factor, was used as an indicative measure of assay robustness and to determine assay performance for each compound’s plate. Dashed lines indicate min/max Z′ values. Screening results are shown in **(C)**. Each point represents the maximum amplitude of individual compounds tested at 10 µM. Grey lines represent mean ± standard deviation (SD).

The experimental design is summarized in Figure 1. Briefly, motile spermatozoa were selected by swim-up and labeled with the fluorescent calcium indicator Fluo-4 a.m. to image changes in [Ca^2+^]_i_. After centrifugation to remove excess dye, Fluo-4 AM loaded cells were distributed in a 384-wells plate to obtain ∼125,000 cells/well. After measuring basal fluorescence, a first injection of compounds was performed in order to evaluate their effect on [Ca^2+^]_i_ alone. This first screen is therefore considered as a screen to search for ‘activators’. Fluorescence was recorded for 12 min during which sperm cells were continuously exposed to compounds. P4 or PGE1 were then added in the same wells to evaluate whether the tested compounds can reduce the ligand-induced activation of CatSper. This second screen was therefore considered to be a search for CatSper ‘inhibitors’.

### Sample collection and preparation

Human semen samples were collected from healthy donors by masturbation after a recommended sexual abstinence period of minimum 2 days. Semen samples were allowed to liquefy at 37°C for 15–30 min. Motile spermatozoa were selected by a ‘swim-up’ procedure as previously described (Strünker et al., 2011). Briefly, 1 mL of semen was deposited in a 45°-inclined 50 mL Falcon tube with 4 mL of Human Tubular Fluid (HTF) medium containing: 93.8 mM NaCl, 4.7 mM KCl, 0.2 mM MgSO_4_, 0.37 mM KH_2_PO_4_, 2.04 mM CaCl_2_, 20.98 mM HEPES, 2.78 mM glucose, 21.4 mM Na-lactate, 4 mM NaHCO_3_, 0.33 mM Na-pyruvate, and the pH was adjusted to 7.35. Motile sperm were allowed to swim at 37°C for 1 hour. The supernatant was collected in 15 mL Falcon tubes and washed twice by centrifugation at 700 x g for 20 min. Sperm were supplemented with 3 mg/mL HSA. Written informed consent was obtained from all donors prior to donation and approved by the Cantonal Ethics Committee of the State of Geneva (CCER #14-147).

### High throughput measurements of changes in [Ca^2+^]_i_

Cells were loaded with the fluorescent Ca^2+^ indicator Fluo-4 AM at a final concentration of 2.5 µM for 30 min at 37°C. Excess dye was washed via centrifugation at 700 x g for 20 min and sperm were resuspended in HTF medium to obtain a final concentration of 6.25 x 10^6^ cells/mL. 20 μL of cells (corresponding to ∼125,000 cells) loaded with Fluo-4 a.m. were dispensed in a 384-wells plate (Corning, NY, United States) to allow fluorescence measurement. The dye was excited at 480 nm, and emission was recorded at 530 nm. The change in [Ca^2+^]_i_ was assessed simultaneously in all wells using a Functional Drug Screening System (FDSS/μCELL) (Hamamatsu Phototonics K.K., Yokohama, Japan) at 37°C. Basal fluorescence was first measured for 30 s. A volume of 5 µL of compounds was then added to obtain a final concentration of 10 µM and P4 or PGE1 were used as positive control at a final concentration of 2 µM corresponding to a value close to saturation (EC_80_). P4 and PGE1 were tested at a near-saturating concentration in the HTS in order to evaluate a potential additive or synergistic effect induced by the compounds. Non-specific CatSper inhibitor MDL 12330A was used as a negative control at final concentration of 20 µM while DMSO was used as a vehicle control. For alkalization-induced Ca^2+^ measurement, NH_4_Cl was used as a positive control at a final concentration of 10 mM. Fluorescence was recorded for a total of 16.5 min: 30 s before injection, 12 min after the first injection of the tested compounds, and 4 min after the second injection of either P4 or PGE1. The Prestwick library was tested in technical duplicate. Ca^2+^ response curves were normalized using the following formula $\frac{\left(F-F_{0}\right)}{F_{0}}\times 100$ , where *F*_*0*_ is the average of the basal fluorescence prior to the addition of the compound of interest and *F* corresponds to the fluorescence value at each time point. The ∆F/F_0_ of the vehicle control (DMSO) was subtracted from all the other values to adjust for dilution and pipetting artifacts. The values of all compounds were normalized to their respective controls.

### Assessment of acrosome reaction

The evaluation of acrosome reaction in live human sperm cells was performed using a modified version of a previously described method (Zoppino et al., 2012). Briefly, swim-up recovered motile spermatozoa were allowed to capacitate for 4 h at 37°C in capacitating media (HTF+) containing: 93.8 mM NaCl, 4.7 mM KCl, 0.2 mM MgSO_4_, 0.369 mM KH_2_PO_4_, 2.04 mM CaCl_2_, 20.98 mM HEPES, 2.78 mM glucose, 21.4 mM Na-lactate, 25 mM NaHCO_3_, 6.6 µM Na-pyruvate, and the pH was adjusted to 7.35. Capacitated sperm were then incubated for 30 min with 5 μg/mL *Pisum sativum* agglutinin conjugated with fluorescein isothiocyanate (PSA-FITC) and 10 μg/mL Hoechst-33342. Samples were centrifuged at 700 x g for 10 min at room temperature to remove excess dye and resuspended in HTF+. Sperm were then incubated for 45 min in the absence (DMSO) or presence of the steroids at 10 μM, or positive control P4 at 10 µM. The ionophore A23187 was also used as a positive control and incubated with sperm at 2 µM for 30 min only. PI (0.5 μg/mL) was then added to all samples which were analyzed using BDFACSAria. FITC-PSA fluorescence was detected by excitation at 475 nm and emission at 560/35 nm, PI was detected by excitation at 530 nm and emission at 675/75 nm and Hoechst was detected by excitation at 346 nm and emission at 460 nm. Hoechst-positive and PI-negative cells were classified as live cells. Doublet exclusion was performed by two-dimensional dot plot analysis of forward-scatter width (FSC-W) versus forward-scatter height (FSC-H), and side-scatter width (SSC-W) versus side-scatter height (SSC-H) on the pre-selected singlets. Data were collected from 40,000 PSA-FITC negative events per sample to define the sperm population. Based on the selected population, PI-negative and PSA-FITC-positive cells were selected as live acrosome reacted cells, whereas PI-negative and PSA-FITC-negative cells were selected as live acrosome intact cells.

### Sperm penetration in viscous media

The ability of sperm to penetrate in viscous media similar in viscosity to that found in the female reproductive tract was assessed using the modified Kremer test, as previously described (Rahban et al., 2021). Briefly, swim-up recovered and capacitated sperm cells at 3 x 10^6^/mL were incubated in the absence (DMSO) or presence of steroids (10 µM), or P4 (5 µM) as a positive control for 1 h at 37°C. A glass capillary (0.2 × 4.0 × 50 mm, CM scientific, UK) was filled with 1% (w/v) methylcellulose (MC, 4000 centipoises cP) prepared with HTF media supplemented with 3 mg/mL HSA, respective compounds (10 µM), P4 (5 µM), or DMSO. The glass capillary tube was then sealed on one end with wax (Vitrex, UK) and added to the sperm cells on the open end. Sperm penetration was assessed after 1 h of incubation at 37°C by counting sperm at 1 cm using a microscope with a ×10 objective.

### Statistical analysis

Data are shown as mean ± standard deviation (SD) with “n” referring to the number of independent experiments performed using sperm samples from ≥3 different donors. Statistical analysis and fitting of dose-response relations were performed using GraphPad Prism 8.1.1 (Prism, La Jolla, United States). To generate sigmoidal curves, the concentrations were converted to their respective log values, and the data were normalized using the following formula: $\frac{\left(100\cdot x\right)}{\left(F_{\max}\right)}$ , where F_max_ is the maximum value of the positive control. A four-parameter Hill equation with a variable Hill coefficient was fitted to generate the EC_02,_ EC_50,_ and IC_50_. Statistical significance between control and treated conditions was evaluated using a one-way analysis of variance (ANOVA), followed by Dunnett´s test. A *p*-value <0.05 was considered significant.

### Pharmacophore and statistical modelling

In order to build a ligand-based model that could be used to screen *in silico* for additional steroid-based compounds with an agonist effect, we started by constructing an initial pharmacophore model and a semi-parametric zero-inflated beta regression model using the 90 steroids identified through the screening of the Prestwick library (see Supplementary Figure S1 for the details on the construction of the models). Given the known importance of different physicochemical properties of compounds for their binding to charged porins in bacteria, including their polarity (Acosta-Gutierrez et al., 2018), we developed a physicochemical property-based filtering criterion relying on a statistical regression model. This model was then used in combination with our pharmacophore model to increase the probability of the *in silico* screening. The regression model assumes a zero-inflated beta distribution to model the agonist effect, which is the dependent variable, while it considers the original physicochemical properties and their second and third-order interactions between them as independent variables. The QikProp tool of the Schrödinger suite (QikProp, Schrödinger, LLC, New York, NY, 2021) was used to calculate the physicochemical properties of each compound. Of the 10 physicochemical properties selected using the Adaptive Lasso procedure (Zou, 2006) and included in the model, seven were identified as significant when considering a confidence level of 1−α=0.95 , most of which were the result of interaction between variables. Out of these variables, only one was an original variable, while the rest of the significant variables were interactions between two or three initially measured properties. To select the most optimal pharmacophore and statistical model combination, we used a blind dataset of 18 compounds whose agonist effect was predicted by the pharmacophore and statistical model prior to their experimental testing. Among the different pharmacophore models considered, the one with the two hydrophobic sites and one acceptor site, combined with a beta zero-inflated model with adaptive-Lasso selected covariates was considered the most optimal (Supplementary Figure S1). In particular, the combination of the two models was able to identify five true positives and one false positive out of the six compounds. The results of the blind test demonstrated that the combination of the two models was crucial to achieving a good prediction ratio, as considering either model alone led to several false positives. Using this combination of models, we screened *in silico* the GeDB (*n* = 187), ChemDiv (*n* = 498), and ZINC (*n* = 98) libraries after querying only for compounds with a steroidal core. For all the compounds screened virtually, 30 additional steroids were tested: the top 30 with the highest pharmacophore fitting score and predicted agonist effect, and five that were expected to have no effect but were included for further validation of the model were purchased and tested experimentally.

## Results

### The Prestwick library screen and confirmation of hits by dose-response

**FIGURE 2:**
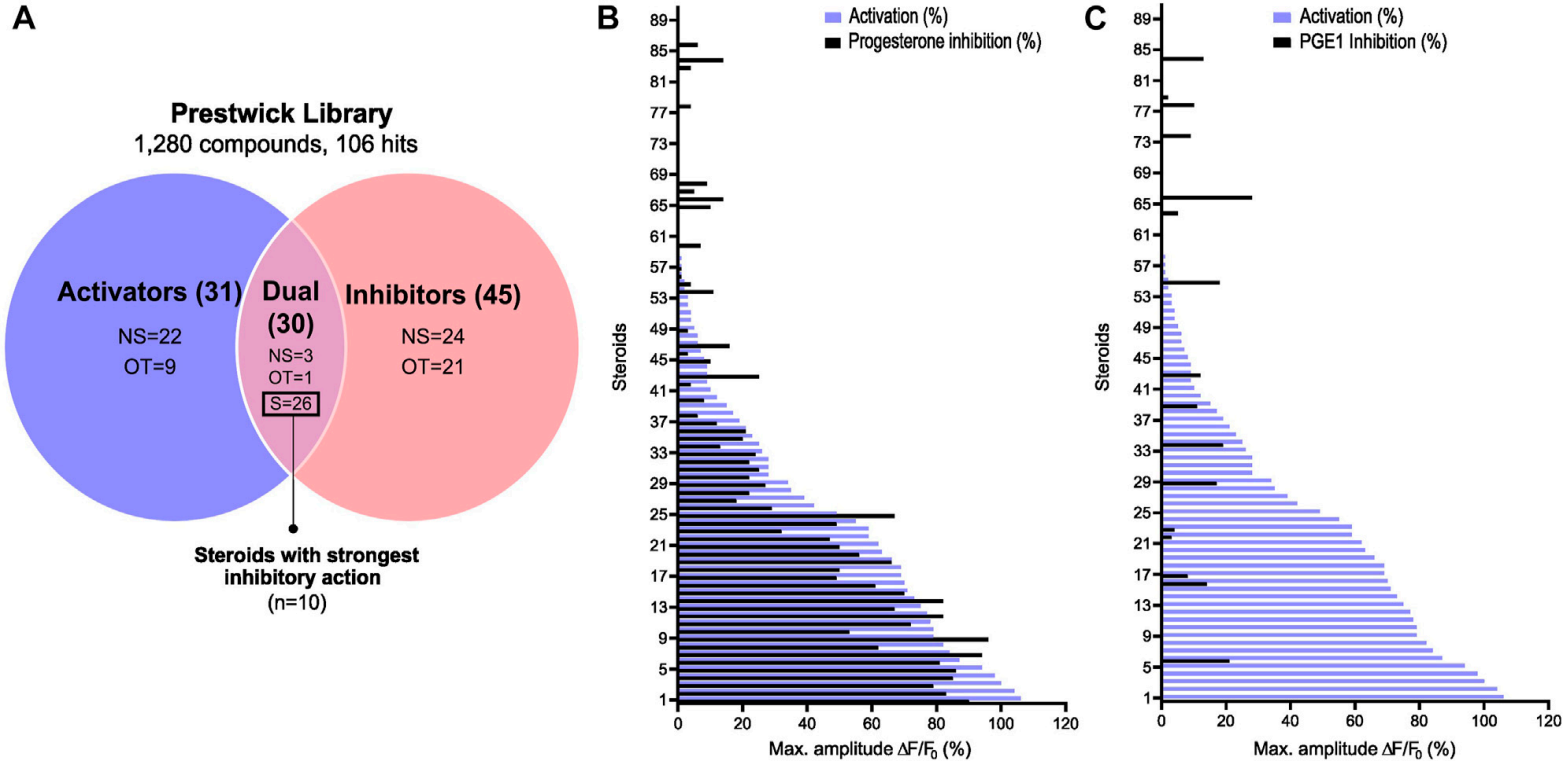
Steroid screening results and steroid selection workflow. **(A)** Venn diagram showing the selection of steroids. A total of 10 steroids with the most potent activating and P4-antagonizing activity were selected. NS: non-steroids; S: steroids; OT: off-targets. Graphical representation of the 90 steroids for their ability to increase the [Ca^2+^]_i_ and inhibit in **(B)** the P4- and in **(C)** the PGE1-induced Ca^2+^ response. Data are expressed as the mean percentage of maximal amplitude ∆F/F0, triggered by each steroid at 10 µM and normalized to P4 response at 2 μM, before (activation, purple) or after the addition of P4 or PGE1 (inhibition in black).

To evaluate the potential effect of synthetic and physiological compounds including steroids on Ca^2+^ influx, we screened the Prestwick library which consists of 1,280 compounds from various therapeutic classes. Our HTS assay was designed to screen for compounds that can induce a Ca^2+^ influx (screen 1, activators) and/or can alter the P4- and PGE1-induced Ca^2+^ influx (screen 2, inhibitors) (Figure 1A). Compound plates were validated with a Z′-factor >0.4 (Figure 1B). Hit selection was based on an arbitrary cutoff of 30% activation and/or inhibition compared to the controls (Figure 1C). We identified 106 (8.3%) hits, of which 31 were putative activators, 45 were putative inhibitors, and 30 had a dual effect (Figure 2A). Interestingly, the compounds with dual activating and inhibiting effects were mostly steroids. All 26 of them reduced the P4- but not PGE1-induced Ca^2+^ influx (Figure 2B, C). The hit steroidal compounds were validated by dose-response curves (DRC) in technical duplicates and biological triplicates and their EC_50_s as well as their IC_50_s for the P4-induced response were measured (Supplementary Table S1). Interestingly, of the 90 steroids in the Prestwick library, more than half (53%) were able to both activate and inhibit P4-induced Ca^2+^ influx in human sperm (Figure 2B). In contrast, inhibition of PGE1-induced Ca^2+^ influx was rarely observed with these steroids (Figure 2C). For the next part of this study, the 10 steroids with the strongest dual activation and inhibition potential were selected for further investigation. These were the synthetic steroids stanozolol, estropipate, dydrogesterone, medrysone, and the natural steroids dehydroepiandrosterone acetate (DHEA), testosterone, epiandrosterone, nandrolone, pregnenolone, deoxycorticosterone.

### Structurally diverse steroids modulate human CatSper

**FIGURE 3:**
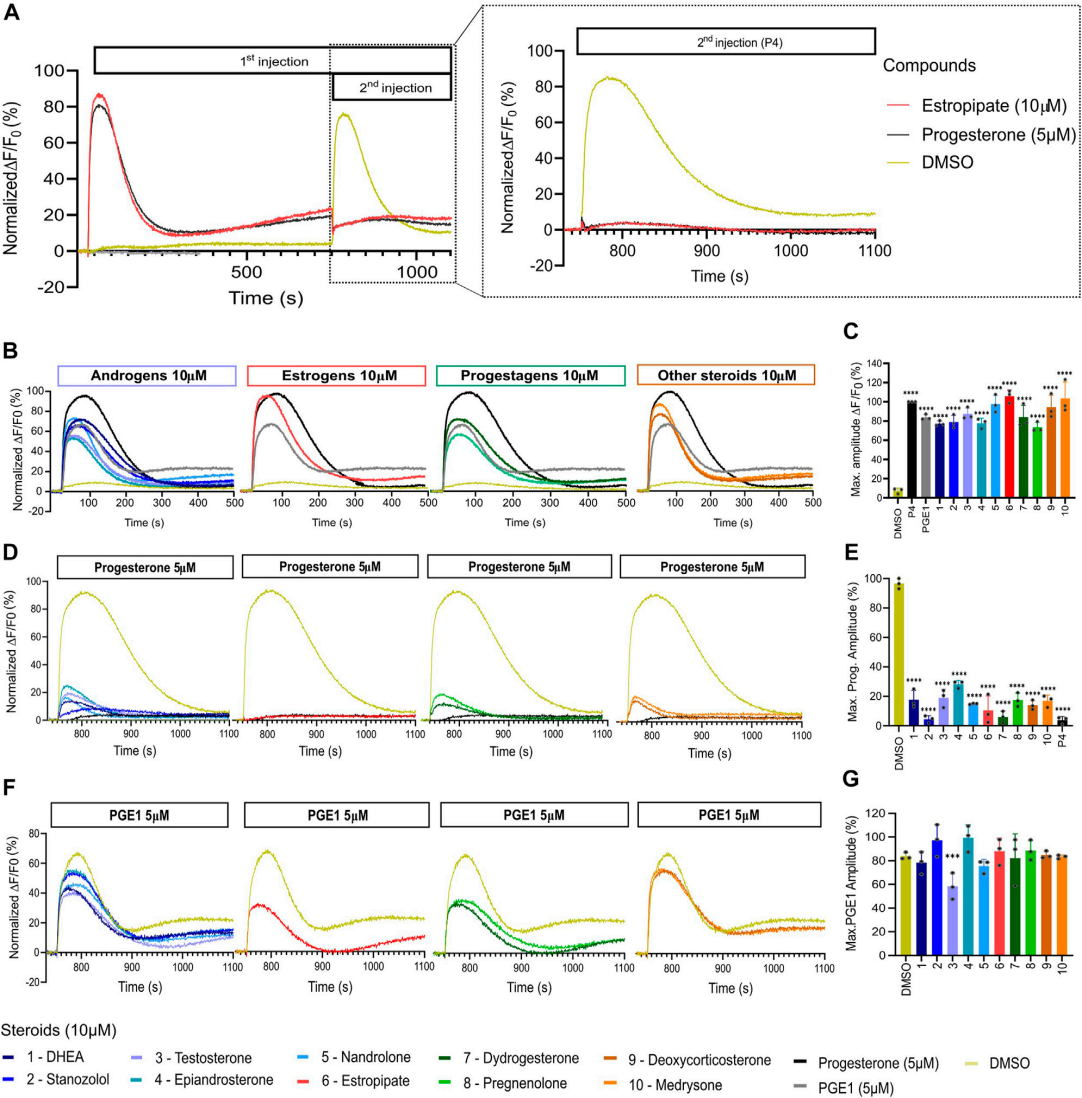
Representative curves of [Ca^2+^]_i_ in human sperm cells induced by diverse steroids. **(A)** Representative curves of Ca^2+^ influx before and after the first addition of steroids alone (10 µM), buffer (DMSO 0.5%) or positive control (P4 or PGE1 - 5 µM) and a second addition to assess the impact of steroids on the P4 or PGE1 response. **(B)** Representative Ca^2+^ signals evoked by each of the 10 steroids at 10 µM (P4 and PGE1 at 5 µM were used as corresponding positive controls). **(C)** Bar plots representing the maximum amplitude of the increase in [Ca^2+^]_i_ shown in B normalized to DMSO (*n* = 3). **(D)** Representative Ca^2+^ signals evoked by P4 (5 µM) after 12 min incubation of each of the 10 steroids. **(E)** Bar plot showing the maximum amplitude evoked by P4 in D normalized to DMSO (*n* = 3). **(F)** Representative Ca^2+^ signals evoked by PGE1 (5 µM) after 12 min incubation of each of the 10 steroids. **(G)** Bar plot showing the maximum amplitude evoked by PGE1 in F normalized to DMSO (*n* = 3). p(***) <0.001; p(****) <0.0001.

**FIGURE 4:**
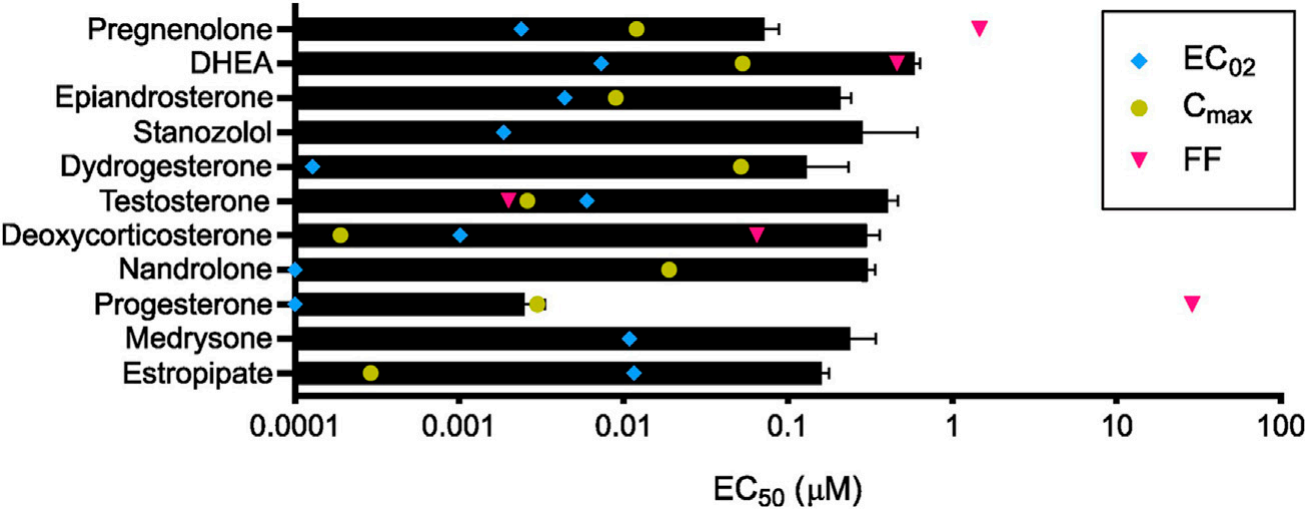
Steroids and their pharmacological concentrations. Calculated *in vitro* half maximal effective concentration (EC_50_, black) compared with reported maximum serum concentration (C_max_, yellow), average concentration in follicular fluid (FF, pink), and calculated minimum effective concentration (EC_02_, blue) (*n* = 3). C_max_ was not found to be reported in the literature for medrysone and stanozolol as well as the FF concentration for epiandrosterone, stanozolol, dydrogesterone, nandrolone, medrysone, and estropipate.

The 10 selected steroids were classified into 4 classes: androgens, estrogens, progestagens, and ‘others’, and they all induced an increase in [Ca^2+^]_i_ similar to P4 with estropipate inducing the highest increase in [Ca^2+^]_i_ (Figures 3A, D). All 10 steroids were tested in dose-response experiments and revealed a dose-dependent increase in [Ca^2+^]_i_ (Supplementary Figure S2). More specifically, estropipate, pregnenolone, and dydrogesterone induced a dose-dependent increase in [Ca^2+^]_i_ with effective concentrations as low as 10 nM. Stanozolol and nandrolone also induced a 30% increase in [Ca^2+^]_i_ at low concentrations in the nanomolar range (Supplementary Figure S2). EC_50_ values ranged from 0.073 µM for pregnenolone to 0.5 µM for DHEA (Table 1). To determine the lowest activating concentration, EC_02_ was calculated for all steroids, and compared with the reported maximum concentration found in blood serum (C_max_) and the reported average concentration of steroids found in the follicular fluid (FF) (Figure 4). This comparison highlighted that the effective concentration at which progesterone, pregnenolone, dydrogesterone, epiandrosterone, nandrolone, and DHEA activate CatSper *in vitro* is within the biological range of their concentration measured *in vivo*. In the case of nandrolone, progesterone, and dydrogesterone, the EC_02_ values were more than 100 times lower than the C_max_ reported value in the literature (Table 1; Figure 4). All steroids tested significantly reduced the P4 response with IC_50_ values ranging from 0.19 µM to 1.75 µM (Supplementary Figure S2; Table 1). This indicates that P4 is the most potent steroid tested. Interestingly, a strong correlation was observed between the steroids inducing the highest increase in [Ca^2+^]_i_ and the ones that reduce the P4 response the most potently (Supplementary Figure S3). This decrease was not class-specific, suggesting that different functional groups can be accommodated in the binding site. Steroids did not alter the PGE1-induced increase in [Ca^2+^]_i_ suggesting that steroids act on the same binding site as that of P4 but not that of PGE1 (Figures 3C, F).

**TABLE 1:** Summary table of EC_50_ and IC_50_ values of the ten selected steroids. All EC_50_ and IC_50_ were generated following dose-response experiments in the absence (DMSO) or presence of steroids ranging from 90 μM to 0.3 nM EC_50_/IC_50_ units are in µM. ND: No data available.

Common name	Chemical structure	Steroid class	EC_50_ (±SD)	IC_50_ (±SD)	Maximum serum level	Physiological presence	Common use	References
Progesterone	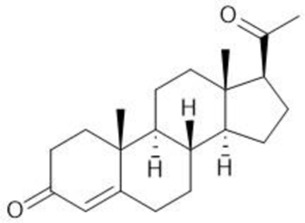	Progestagen	0.003 (±0.001)	0.006 (±0.002)	3 nM (male)	Yes	Birth control	Löfgren and Bäckström (1990)
					60 nM (female)			
Stanozolol	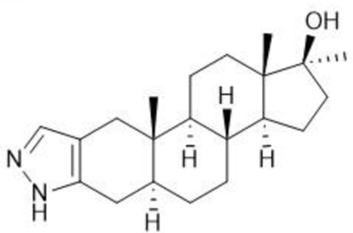	Androgen	0.285 (±0.328)	0.194 (±0.045)	ND	No	Anabolic-Androgenic Steroids (AAS)	ND
Pregnenolone	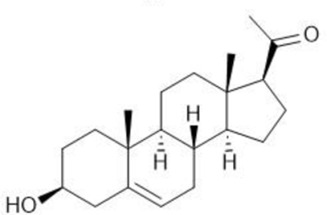	Progestagen	0.073 (±0.016)	0.199 (±0.008)	12 nM	Yes	Endometriosis	Hill et al. (1999)
Dydrogesterone	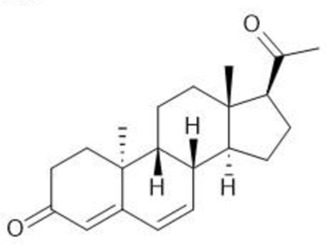	Progestagen	0.130 (±0.103)	0.245 (±0.042)	52 nM	No	Luteal insufficiency infertility	Abdel-Hamid et al. (2006)
Estropipate	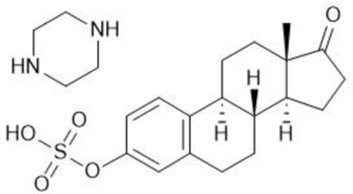	Estrogen	0.161 (±0.017)	0.288 (±0.010)	0.29 nM	No	Ovarian failure	Barnes and Levrant (2007)
Medrysone	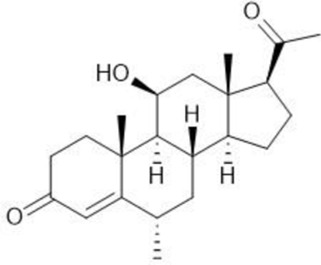	Corticosteroid	0.240 (±0.103)	0.569 (±0.275)	ND	No	Anti-inflammatory	ND
Deoxycorticosterone	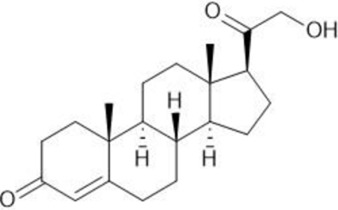	Mineralocorticoid	0.302 (±0.062)	0.523 (±0.081)	0.19 nM	Yes	Adrenocortical insufficiency	Oddie et al. (1972)
Epiandrosterone	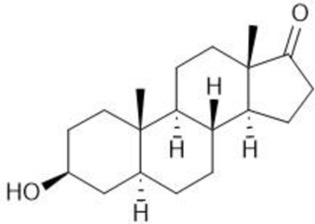	Androgen	0.209 (±0.033)	0.628 (±0.102)	9 nM	Yes	AAS	Lewis et al. (1997)
Nandrolone	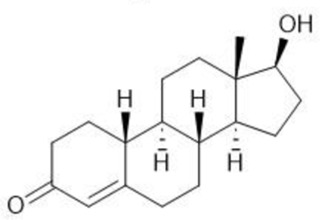	Androgen	0.306 (±0.033)	0.711 (±0.109)	19 nM	No	AAS; Anemia	Bagchus et al. (2005)
Testosterone	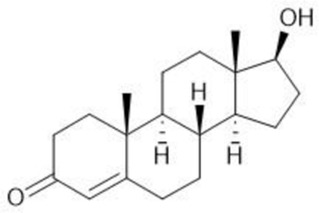	Androgen	0.407 (±0.062)	1,107 (±0.157)	11.6 nM	Yes	Sexual dysfunction (men); hot flashes (women)	Groepenhoff et al. (2021)
Dehydroisoandosterone 3-acetate (DHEA)	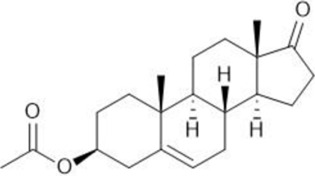	Androgen	0.593 (±0.043)	1,745 (±0.488)	53 nM	Yes	Depression, Supplement	Habib et al. (2021)

### Steroids compete for the same binding site as progesterone

**FIGURE 5:**
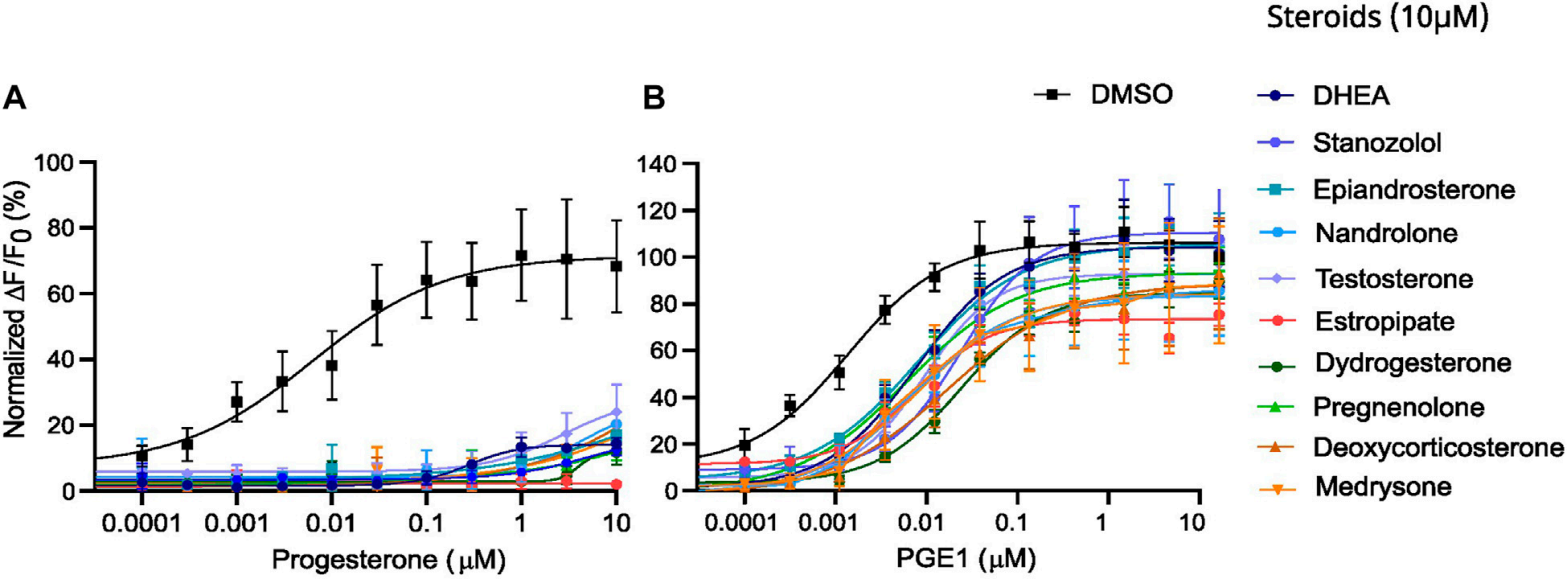
Desensitization experiments with increasing doses of P4 and PGE1 in the presence of a fixed dose of steroids. Dose-response curves of **(A)** the P4-induced and **(B)** the PGE1-induced Ca^2+^ response in the absence (DMSO) and presence of steroids at 10 µM. Data are presented as the mean of 3 independent experiments with error bars representing the standard deviation (SD).

To determine whether the strong inhibitory effect of the 10 steroids on P4-induced Ca^2+^ influx was due to competitive binding, we performed cross-desensitization experiments by challenging pre-incubated sperm cells with steroids at 10 µM with increasing doses of P4 (Figure 5A). The P4-induced increase in [Ca^2+^]_i_ was reduced significantly by the presence of the 10 steroids tested. In contrast, the same experiment performed with increasing doses of PGE1 revealed a slight shift in the EC_50_ without a decrease in maximum response, indicating that steroids reduce the potency of PGE1 but not its efficacy. (Figure 5B).

### Steroids suppress pH-induced activation of CatSper

**FIGURE 6:**
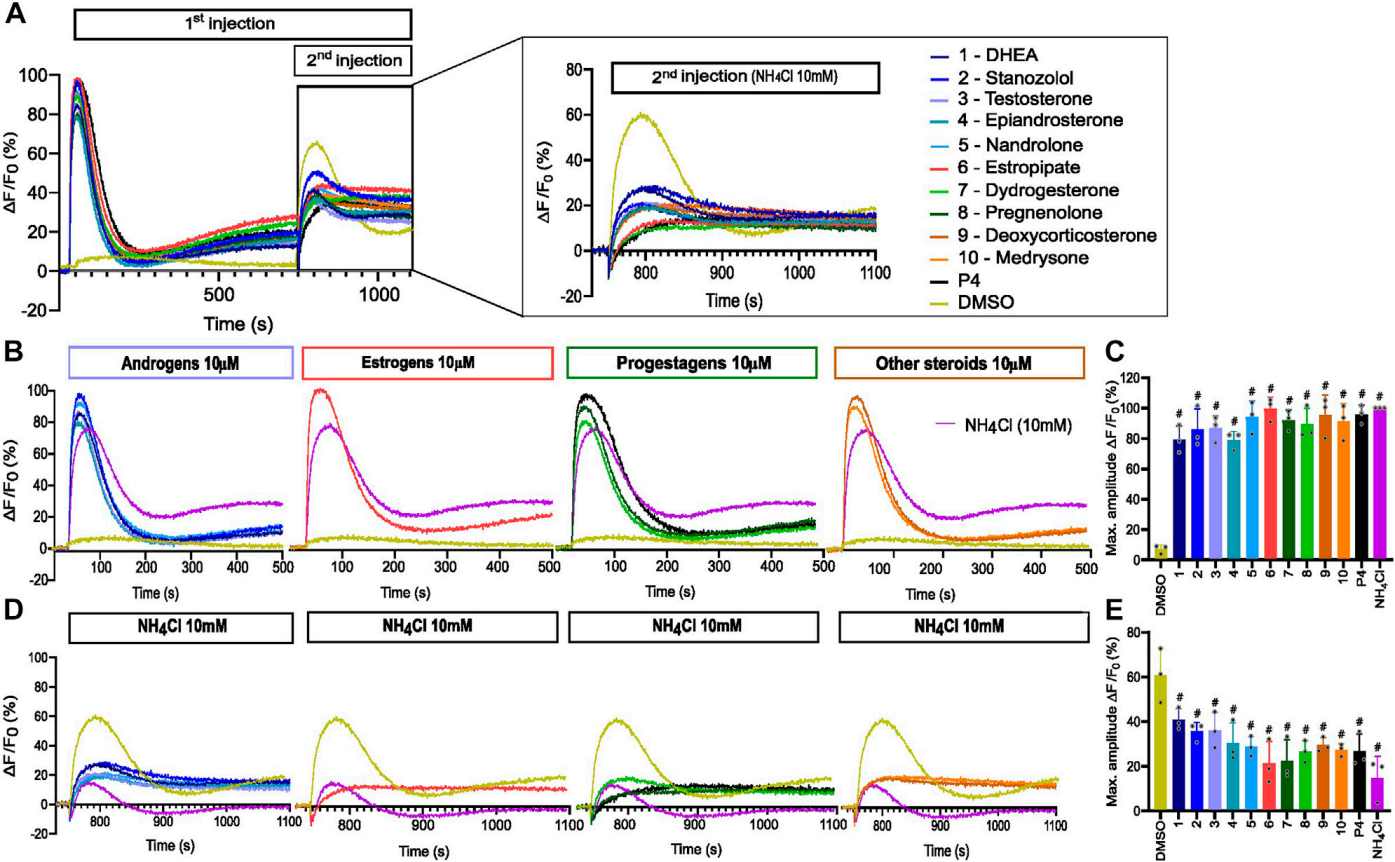
Alkaline-evoked [Ca^2+^]_i_ signals in the presence of steroids. **(A)** Representative curves of Ca^2+^ influx before and after the first addition of steroids alone (10 µM), buffer (DMSO 0.5%), or positive control NH_4_Cl (10 mM) and a second addition to assess the impact of steroids on the NH_4_Cl-induced response. **(B)** Representative curves of Ca^2+^ signals induced by steroids at 10 µM alone and positive control NH_4_Cl at 10 mM. **(C)** Bar plots representing the maximum amplitude of Ca^2+^ signals in B (*n* = 3). **(D)** Ca^2+^ influx evoked by NH_4_Cl (10 mM) after 12 min incubation of spermatozoa with steroids at 10 µM. **(E)** Bar plot of maximum amplitude of Ca^2+^ signals in D (*n* = 3), p(#) <0.0001.

Besides ligand activation, CatSper can be weakly activated by changes in membrane voltage or alkalization of intracellular pH (pH_i_) (Brown et al., 2019; Wang et al., 2021). Therefore, the 10 selected steroids were evaluated for their ability to affect CatSper-mediated Ca^2+^ influx induced by intracellular alkalization via the weak base NH_4_Cl. All tested steroids almost completely suppressed the Ca^2+^ signals evoked by NH_4_Cl at 10 mM (Figure 6). We also measured changes in pH_i_ in the presence of the 10 steroids. None of the steroids induced an increase in intracellular pH (Supplementary Figure S4), indicating that CatSper activation by steroids is not triggered by the increase in pH_i_.

### Structure-activity relationship of agonist steroid compounds

**FIGURE 7:**
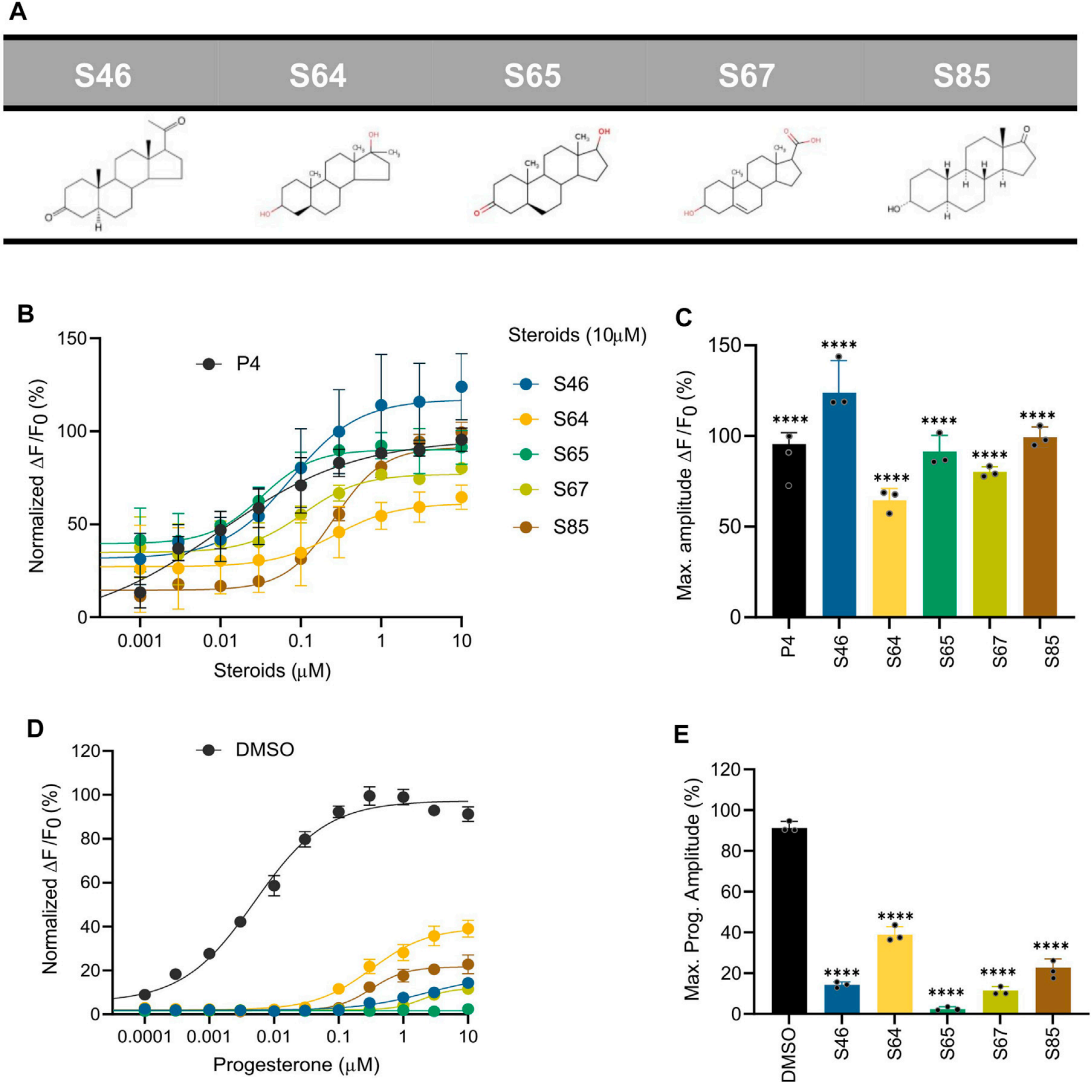
Ca^2+^ response and P4-induced response in the presence of steroids predicted by pharmacophore and statistical modeling. **(A)** Chemical structure of the top five predicted steroids selected from the pharmacophore and statistical regression models. **(B)** Concentration-response curves comparing the potencies of the steroids with P4. **(C)** Bar plot of the maximum signal amplitude (∆F/F_0_ (%)) of the steroid-induced Ca^2+^ response in **(B, D)** Concentration-response curves comparing the inhibition of P4-induced [Ca^2+^]_i_ influx by the top five predicted steroids at 10 μM, normalized to DMSO. The generated EC_50_s were the following: EC_50_ (P4): 0.005 µM**,** EC_50_ (S46): 2.254 µM, EC_50_ (S64): 0.322 µM; EC_50_ (S65): NA; EC_50_ (S67): 2 μM; EC_50_ (S85): 0.32 µM. **(E)** Bar plot of the maximum signal amplitude shown in D normalized to DMSO (n = 3). All steroids induced a significant inhibition of the response elicited by P4. Data are plotted as the mean of three independent experiments with error bars representing the SD and expressed as a percentage of the response elicited by P4 (10 µM) or DMSO (0.5%); p(****) <0.0001.

**FIGURE 8:**
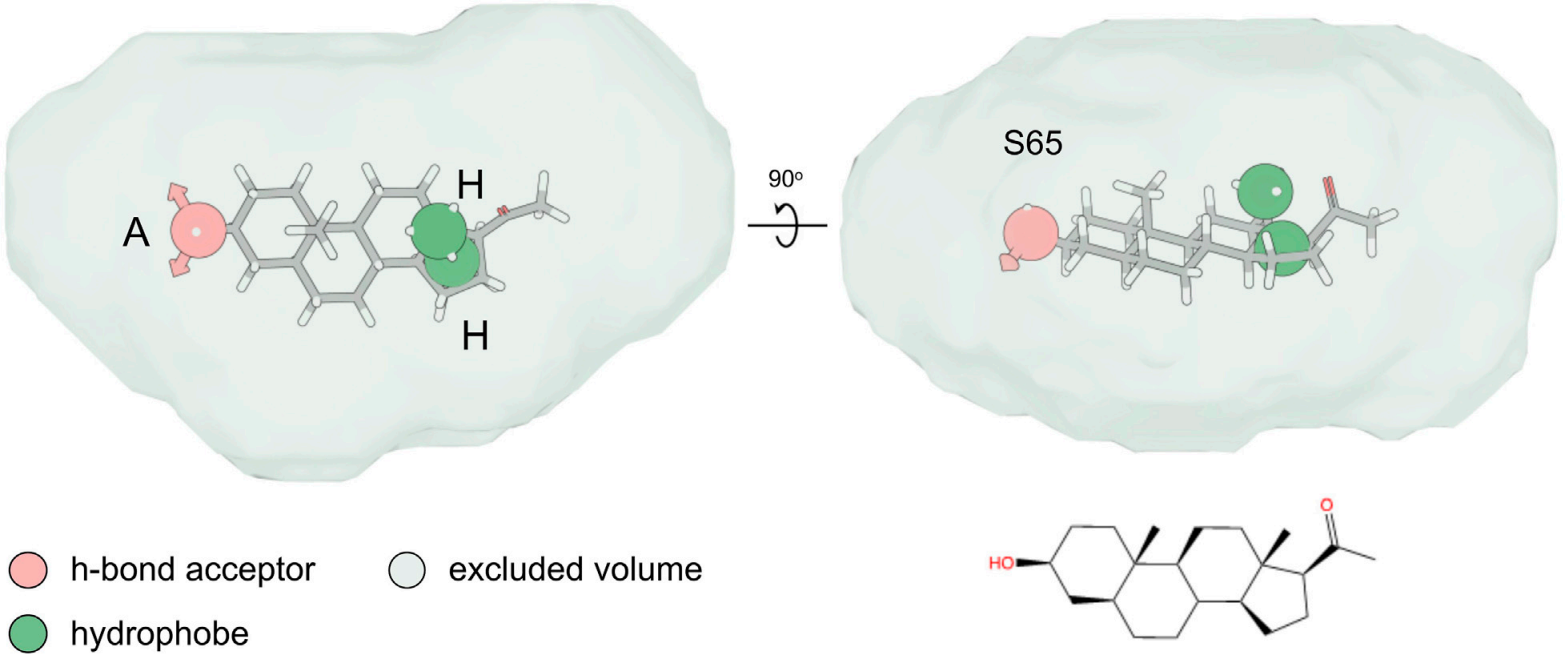
Pharmacophore hypothesis. Top and side view of the fitting of compound S65 into the pharmacophore hypothesis depicting the minimum pharmacophore features that a ligand needs to satisfy for it to interact non-covalently with its receptor. The hydrogen-bond acceptor (“A”) and hydrophobe (“H”) pharmacophore features are shown as spheres, while the excluded volume that was considered based on the shape of the agonist compounds is shown as a surface.

In the absence of a resolved structure for human CatSper, we used pharmacophore modelling and machine learning to search *in silico* for additional steroid compounds with a strong activating effect as well as define the steroid structure-activity relationship (SAR) responsible for the CatSper activation. We first constructed a pharmacophore model for the steroids with agonist activity that we identified in our initial screening (Figures 2B, C), and then combined them with a statistical regression model capable of relating their physicochemical properties to their activity profile. After validating the ability of our combined model to identify compounds that could increase the Ca^2+^ influx against a blind dataset, we used it to screen 783 compounds *in silico*. Of the 20 top compounds that we selected and tested experimentally, 11 showed activity in the high nanomolar range (Supplementary Table S1). Of particular interest are the synthetic steroids S46 and S65 (Figure 7A). These two compounds potently increased [Ca^2+^]_i_ in human sperm (EC_50_ of 32 nM and 73 nM, respectively) at concentrations close to P4 (EC_50_ of 2.5 nM) (Figures 7B, C). These two compounds also significantly decreased the response to P4 (IC_50_ of 46 nM and 138 nM, respectively) (Figures 7D, E). Among the different physicochemical properties that we considered for the construction of the statistical regression model to predict the agonist effect of new steroids, we found a significant positive correlation between the predicted IC_50_ value for human Ether-à-go-go-Related Gene (hERG) K^+^ channel block (QPlogHERG) and the number of amide groups and the square of the dipole moment divided by the molecular volume (dip^2^/V). This finding is not unexpected as dip^2^/V is a crucial term in the free energy of solvation of a dipole with volume V. Additionally, dip^2^/V and the octanol/water partition coefficient (QPlogP_o/w_), as well as dip^2^/V and the y-component of the dipole moment, were found to be significantly positively correlated with the dependent variable, in this case, the agonist effect. The coefficients of the properties used in the estimated model can be found in Supplementary Table S2. From our model, it is clear that the desolvation of the steroids and their dipole moment in the direction of the electric field inside the channel with respect to their volume are key properties that need to be fine-tuned when designing new steroid analogues that target CatSper. To investigate the SAR of steroid activation of the CatSper channel, we evaluated a wide range of steroids with different functional groups in each of the steroid rings for their ability to induce Ca^2+^ influx in human sperm. Our results showed that the CatSper channel was activated by multiple steroids with various structural modifications. Structural analysis of the steroids provides useful insights into substitutions that can improve the potency of P4 analogues (Figure 8). In particular, various substitutions on the A-ring could be tolerated, including a pyrazole ring (stanozolol, Supplementary Table S1), resulting in moderate CatSper activators. Interestingly, the removal of the double bond present on the A-ring of P4 also increased the agonist activity of some compounds such as pregnenolone, S67, S85, and S45 (Figures 7B, C; Supplementary Table S1). This suggests that some flexibility in this ring may facilitate stronger interactions with the channel residues. In agreement with previous reports (Carlson et al., 2022b), methylation at the C19 position on the B-ring does not appear to be important for the activity, whereas our results indicate that methylation at the C7 position on the same ring can be tolerated. Any significant substitution on the C-ring rendered the steroids inactive or unable to bind at all. Only steroids with a hydroxyl group at the C13 or C14 position seemed tolerable in our dataset. Finally, bulky aliphatic substitutions at the C17 position of the D-ring led to a dramatic decrease in agonist activity, but small substitutions with hydrogen bond donors had a modest activating effect (deoxycorticosterone, stanozolol, norgestimate, Supplementary Table S1), suggesting that this part of the steroid may play a key role in stabilizing binding and thus activating CatSper. Notably, structural modifications on the D-ring have been reported to convert agonists to antagonists (Carlson et al., 2022b) and, therefore, this region can be exploited in the design of potent steroidal antagonists of CatSper (Supplementary Figure S1).

### Steroids do not affect the acrosome reaction but Stanozolol and estropipate increase sperm penetration in viscous media

**FIGURE 9:**
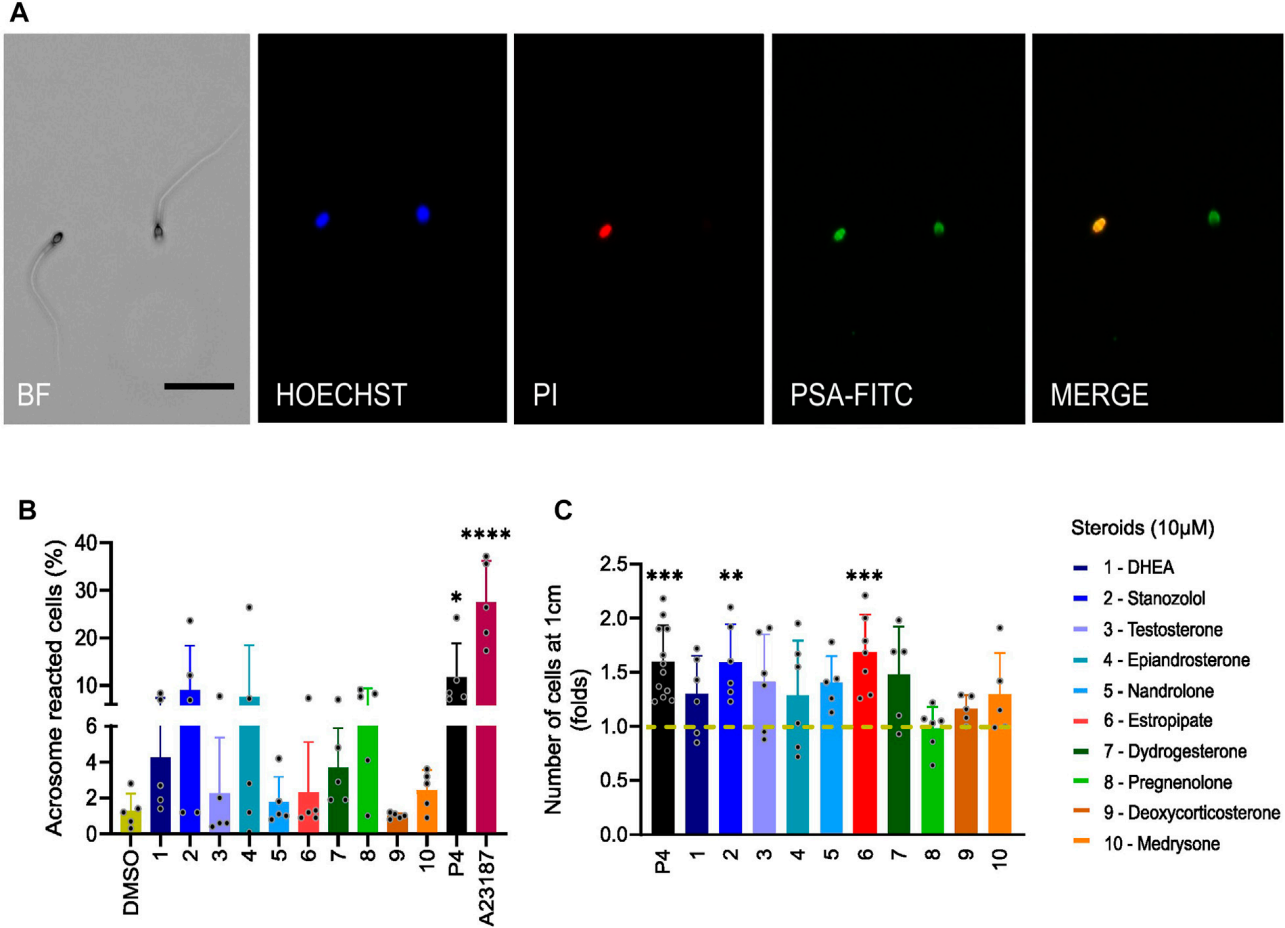
Sperm acrosome reaction and penetration in viscous media induced by steroids. **(A)** Images of fluorescently labeled spermatozoa obtained by bright field (BF) and fluorescence microscopy. Sperm were incubated for 30 min with *Pisum sativum* agglutinin-fluorescein isothiocyanate (PSA-FITC), Hoechst 33342 and propidium iodide (PI). Merge indicates the overlap of fluorescence images between PSA-FITC and PI-labeled spermatozoa. Scale bar = 50 µm. **(B)** Percentage (±SD) of live acrosome-reacted sperm in the presence of tested steroids (10 µM), P4 (10 µM), or ionophore A23187 (2 µM) used as positive controls, normalized to DMSO (*n* = 5). **(C)** The number of sperm cells that were able to penetrate a glass capillary tube filled with 1% methylcellulose was assessed after incubation of sperm with steroids (10 µM), or P4 (5 µM) or DMSO for 1 h. The number of cells is normalized to DMSO (shown in dashed mustard line) and results are presented in fold change. Stanozolol and estropipate significantly increased the number of sperm cells that can penetrate in viscous medium (*n* > 5). p(*) < 0.05; p(**) <0.01; p(***) <0.001; p(****) <0.0001.

We finally investigated the effect of the 10 selected steroids on the acrosome reaction (AR) in human sperm (Figure 9A). All steroids showed similar results to negative control DMSO except stanozolol, epiandrosterone, and pregnenolone. These steroids induced acrosome reaction in more than 10% of the cells, but this increase was not significant because of the large variation in response among donors. P4 only slightly increased the AR in consistence with previously published data (Baldi et al., 1991; Prajapati, et al., 2022). We also assessed sperm penetration in a viscous medium using a modified Kremer’s test (Figure 9B). At a distance of 1 cm, the majority of the steroids tested increased the number of sperms that can penetrate in viscous media, although only stanozolol and estropipate induced a significant increase in cell number similar to P4. In addition, we evaluated the sperm motility in the presence and absence of the selected steroids at 10 µM using Computer Assisted Sperm Analysis (CASA) (Supplementary Figure S5). Incubation of spermatozoa with the selected steroids for 1 hour did not alter total, progressive, and hyperactive motility (Supplementary Figure S5). Cell viability was also assessed by flow cytometry of PI incubated cells (Supplementary Figure S6). Our results show that steroids at 10 µM were not cytotoxic.

## Discussion

CatSper is the main entry point for Ca^2+^ ions in human spermatozoa, but it is also a promiscuous channel that can be modulated by a wide variety of natural and synthetic molecules (Spehr et al., 2003; Tavares et al., 2013; Schiffer et al., 2014; Miller et al., 2016; Rehfeld et al., 2016; Birch et al., 2021; Jeschke et al., 2021; Rahban et al., 2021). To investigate the impact of diverse chemical structures on [Ca^2+^]_i_, we developed a HTS Ca^2+^ influx assay in human sperm. We screened 1,280 approved and off-patent drugs from the Prestwick chemical library for their potential to either induce a Ca^2+^ influx or to alter the P4- and PGE1-induced response. We show that a wide range of pharmaceutical compounds belonging to the steroid class, both natural and synthetic, are capable of acting on CatSper. Using a pharmacophore and statistical modelling, we screened *in silico* for steroids that can activate the channel and better understand the molecular characteristics of the steroids that promote CatSper activation.

A recent study on 15 steroids present in the follicular fluid showed that these steroids cause a rapid increase in Ca^2+^ influx in human sperm via CatSper (Jeschke et al., 2021). The classical Ca^2+^ response was abolished in CATSPER2^−/−^ spermatozoa for all 15 steroids, demonstrating that the response depends on Ca^2+^ influx via CatSper. Here, we show that the ability of the steroids to activate CatSper is highly variable, with some steroids activating CatSper at low nanomolar concentrations while others are completely ineffective in inducing a Ca^2+^ response. In particular, 10 steroids had a strong dual activating and inhibiting effect on CatSper and were therefore selected for in-depth analysis of their action. These steroids were either synthetic (stanozolol, estropipate, dydrogesterone, medrysone) and/or physiological (dehydroepiandrosterone acetate - DHEA -, testosterone, epiandrosterone, nandrolone, pregnenolone, deoxycorticosterone) and were all evaluated for their action on P4-, PGE1-, pH- activation of human CatSper. In addition to cross-desensitization experiments, our results showed that all 10 steroids directly modulate the CatSper channel and compete for the same binding site as P4, but not PGE1. These observations are consistent with several scientific reports indicating that numerous steroids such as testosterone, hydrocortisone, estradiol, pregnenolone, dihydrotestosterone, and estrone act as true agonists that activate CatSper and increase [Ca^2+^]_i_ in human sperm (Blackmore et al., 1990; Blackmore et al., 1996; Luconi et al., 1999; Luconi et al., 2004; Rossato et al., 2005; Brenker R. A. et al., 2018; Brenker S. C. et al., 2018; Rehfeld, 2020; Jeschke et al., 2021). These results also demonstrate that steroids and prostaglandins act on distinct binding sites, as previously described (Lishko et al., 2011; Strünker et al., 2011; Miller et al., 2015; Kaupp and Strünker, 2017; Lishko and Mannowetz, 2018).

Steroids are widely used as medications for a variety of purposes, some on a long-term basis, and have been available for over 50 years (Rice et al., 2017; Kapugi and Cunningham, 2019; Bleecker et al., 2020). Corticosteroids are one of the most commonly prescribed steroids and are often used to reduce inflammation and suppress the immune system in conditions such as asthma, allergic rhinitis, chronic obstructive pulmonary disease, multiple sclerosis, and others (Rice et al., 2017; Ramadan et al., 2019; Bleecker et al., 2020). Synthetic versions of testosterone and estrogen are also used by some athletes (anabolic steroids) and postmenopausal women, respectively (Cole et al., 2019). Our results suggest that steroids such as deoxycorticosterone significantly increased [Ca^2+^]_i_ and reduced more than 86% of the P4 response with an EC_50_ = 0.3 µM and an IC_50_ = 0.5 µM, respectively. Similarly, androgens and estrogens also acted as both potent activators and inhibitors at low nM concentrations. Therefore, an important matter to be addressed here is the physiological significance of the pharmaceutical steroid on CatSper activation. To our knowledge, few previous studies have reported on the concentration of steroids in seminal, oviductal, or follicular fluid. Jeschke *et al.* reported the average concentration of 18 steroids measured in the follicular fluid of eight oocytes from four different gonadotropin-stimulated women. Among the 18 steroids, five were steroids that we tested: progesterone, pregnenolone, deoxycorticosterone, DHEA, and testosterone (Jeschke et al., 2021). Here we show that of the 10 steroids analyzed, pregnenolone, dydrogesterone, epiandrosterone, nandrolone, and DHEA are present in sufficient quantities to activate CatSper with EC_02_ below the reported maximum concentration found in blood serum. We show here that these steroids compete for the same binding site, suggesting that they can bind to CatSper *in vivo* and potentially affect P4 binding and downstream sperm function. For fertilization to occur, it is crucial that sperm functions controlled by [Ca^2+^]_i_ are triggered appropriately and in a timely manner (Publicover et al., 2007). We therefore argue that binding of these steroids to CatSper before the sperm reaches the oocyte could prevent the optimal binding of P4 and thus the fertilization process. This in turn could reduce the fertility of couples. It has been shown on several occasions that a suboptimal P4-induced Ca^2+−^influx is associated with reduced male fertility and that impaired P4-induced calcium signaling is associated with lower rates of IVF success (Falsetti et al., 1993; Krausz et al., 1995; Forti et al., 1999; Williams et al., 2015; Kelly et al., 2018).

Considering that humans may be exposed to a variety of chemicals that can interfere with CatSper, and these molecules may act additively or synergistically with steroidal compounds to affect CatSper-mediated Ca^2+^ signaling (Schiffer et al., 2014; Rehfeld et al., 2016; Brenker R. A. et al., 2018). To this end, further, *in vitro* and clinical studies are needed to assess the levels of EDCs and other relevant steroids in oviductal fluids and to investigate whether synergistic interactions between these compounds may affect the activity of CatSper. It is worth noting that the antagonizing activity of the steroids on the P4-induced response can also be considered relevant in the field of male contraception (Wang et al., 2021; Rehfeld et al., 2022). Indeed, steroids such as stanozolol and dydrogesterone that strongly reduce the P4 response can serve as a starting point in that direction.

Although all selected steroids had a significant impact on CatSper activation or reduction of the P4-induced response, none affected the AR, while stanozolol and estropipate only slightly increased sperm penetration into viscous media. It is well established that P4 acts on CatSper (Lishko et al., 2011; Strünker et al., 2011). However, the downstream effects of this activation on human sperm function, such as the AR and sperm penetration in viscous media have been highly controversial (Baldi et al., 2009; Tamburrino et al., 2014). Recently, Young and others described nine CatSper-deficient men presenting with couples infertility and failure of Medically Assisted Reproduction (MAR) (Young et al., 2023). They showed that loss of CatSper function abolished spontaneous hyperactivation induced by capacitation and P4, but did not affect AR and sperm penetration in viscous media (Young et al., 2023). This suggests that most of the steroids tested here act on CatSper in a similar way to P4.

To provide structural insights into the activation of CatSper by steroids, we used the dataset we generated on over 90 steroids and applied pharmacophore modelling and machine learning to define the structure-activity relationship (SAR) associated with the agonist effect of the tested steroids. Due to the wide range of steroids with different functional groups in each of the steroid rings, we were able to refine the SAR for steroid activation of the CatSper channel and provide useful information on substitutions that may enhance the ability of certain steroids to activate CatSper. Our results show that the A-ring of steroids can tolerate various substitutions, including a pyrazole ring, resulting in moderate CatSper activation. Removal of the double bond on the A-ring was shown to increase the activity of some compounds, suggesting that the flexibility of this ring facilitates stronger interactions with the channel residues. Methylation at the C7 position on the B-ring is tolerated, but any significant substitution on the C-ring renders the steroids inactive or unable to bind. Steroids with a hydroxyl group at the C13 or C14 position appeared to be tolerable, and the bulky aliphatic substitutions at the C17 position of the D-ring led to a dramatic decrease in agonist activity. Our results are consistent with those of previous studies that have examined the chemical structures of steroids to determine their affinity for the unknown binding site of CatSper (Jeschke et al., 2021; Carlson et al., 2022a; Carlson et al., 2022b; Xiang et al., 2022). The use of the SAR information allowed us to identify and test several commercially available synthetic steroids in addition to the steroids we initially screened. Some of these steroids were able to increase [Ca^2+^]_i_ and inhibit the P4-induced response at nM concentrations, close to P4. The reduced response suggests that this combined pharmacophore and physicochemical properties modelling approach is effective and allows the identification of steroids capable of activating CatSper already at very low concentrations. The study from Carlson et al. also investigated the effect of multiple steroids and found that CatSper is activated by a wide range of steroids with diverse structural modifications (Carlson et al., 2022b). They examined the modification of residues throughout the steroid skeleton and found that over 30 steroids are capable of activating CatSper. Interestingly, all modifications reduced potency relative to P4, generally without affecting the maximal extent of Ca^2+^ influx. Here, we have extended this analysis by examining 90 steroidal drugs as well as 30 commercially available synthetic steroids selected by SAR for steroid activation of the CatSper channel. Our data confirm those of Carlson et al. as we found that more than half of the steroids tested can activate CatSper and selectively antagonize P4-induced Ca^2+^ influx. Furthermore, the potency of activation or inhibition of the P4 response by these steroids is always lower than that of P4. Our *in silico*-based screening identified several steroidal compounds with high potencies capable of inducing Ca^2+^ influx and inhibiting P4-induced response at a low concentration within the nM range (compounds S65 and S46 with EC_50_ of 32 nM and 73 nM and IC_50_ of 46 nM and 138 nM, respectively). In contrast, the most potent steroids inhibiting P4-induced response identified by Carlson et al. such as medroxyprogesterone acetate, levonorgestrel, and aldosterone have IC_50_ values two orders of magnitude higher at 6.6 µM, 32.3 µM, and 33.1 µM, respectively.

In conclusion, our results suggest that diverse steroids act directly on CatSper to activate the channel and compete with P4 for its binding site *in vitro*. If bound to CatSper prior to physiological P4-binding, these steroids might impair the fertilization process. The mechanism of action by which these steroids act on CatSper is however complex and further studies are required to elucidate their potential adverse effect *in vivo*. In a research setting, their P4-antagonizing activity might serve as a starting point for the development of male contraceptives.

## Data Availability

The raw data supporting the conclusion of this article will be made available by the authors, without undue reservation.
