# Supplementary material for: The action of physiological and synthetic steroids on the calcium channel CatSper in human sperm
Source: Front Cell Dev Biol. 2023 Jul 20;11:1221578. doi: 10.3389/fcell.2023.1221578 (PMC10397409; doi:10.3389/fcell.2023.1221578)
Supplement: Supplementary file 1 [file DataSheet1.docx]

# Supplementary material


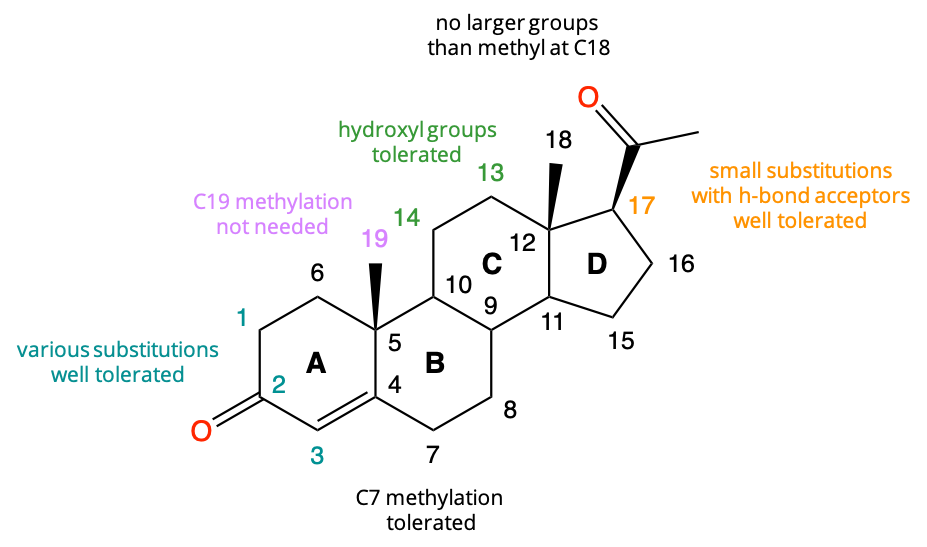


**Supplementary Figure 1: Structure-activity relationships of CatSper activation by steroids.**

*Pharmacophore and statistical model construction*

The pharmacophore and the statistical modelling were constructed as follows. The original dataset of the 90 compounds was pre-processed using the LigPrep pipeline of the Schrödinger suite (LigPrep, Schrödinger Release 2021, LLC, New York, NY, 2021) at pH=7 and the OPLS4 force field to generate all possible enantiomers and conformers of each steroid in the dataset.

For the construction of the pharmacophore model, compounds that induced more than or equal to a 50% increase in [Ca^2+^]_i_ were labelled as active, while the rest were labelled as inactive. **More than** half of the active compounds (n=12) and all of the inactive compounds (n=70) were first aligned using the steroidal core as the common substructure for alignment. The labelled, pre-aligned set was used to develop several pharmacophore hypotheses using the Phase algorithm of the Schrödinger suite (Dixon et al., 2006). For each hypothesis, an excluded volume shell based only on the active compounds was created to gauge the shape of the binding site. In order to evaluate the different pharmacophore hypotheses generated and to select the most optimal ones, we used the DeepDecoy algorithm (Imrie et al., 2021) to generate a series of decoy compounds (n=932), which match the structure and physicochemical properties of the 20 active compounds we provided. The remaining active compounds (n=8) that were not used for the pharmacophore hypothesis development were mixed with the decoy compounds (decoy/active dataset), and we screened this dataset with the different pharmacophore hypotheses we developed. The top three pharmacophore hypotheses that were able to recover all the active compounds in their top ten hits were selected for further evaluation. To design an appropriate statistical model of the expected agonist effect based on the physicochemical properties, we considered 21 physicochemical properties for each of the 90 compounds of the original dataset. We extended the number of covariates by considering interactions up to the second-degree (i.e. combinations of three variables) between the properties, resulting in a design matrix of 1793 covariates. Based on the distribution of the dependent variable, a regression model with zero-inflated beta distribution (Ospina and Ferrari, 2012) appeared to be suitable. We proceeded to the selection of variables with a penalized regression approach, using the Adaptive-Lasso estimator (Zou, 2006) to select the most relevant covariates. As the relationship between some of the covariates and the dependent variable exhibited clear non-linear relationship patterns, we also included non-parametric smoothing splines for some of the selected covariates in addition to the fixed linear effects. Throughout the iterative development of the model, we prevented overfitting by measuring likelihood-based estimators of prediction error, such as the AIC (Akaike, 1974), and empirical measures of predictive performance, such as the root mean square error (RMSE) estimated by a repeated 10-fold cross-validation. The final model was selected to minimize these criteria. Statistical analysis was performed in the R language using the GAMLSS library for the model estimation (Stasinopoulos, 2017).

**A**

**B**


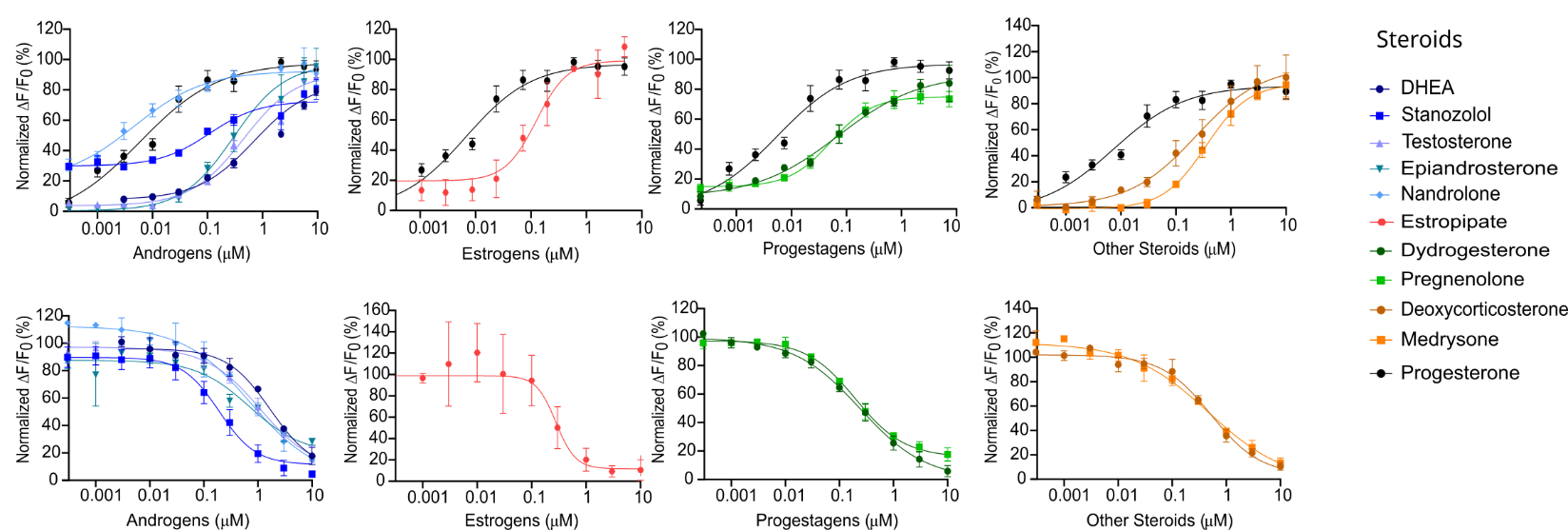


**Supplementary Figure 2: Steroids induce an increase in [Ca^2+^]_i_ and inhibit the P4-induced Ca^2+^ response in human spermatozoa**. (**A)** Dose-response curves of all 10 steroids grouped by steroid classes and compared with the positive control P4 (black curve). (**B)** Dose-response curves comparing the inhibition of P4-induced Ca^2+^ influx by the 10 steroids grouped by steroid classes. Data are plotted as the mean of three independent experiments with error bars representing the SD and are expressed as a percentage of the response elicited by the saturating concentration of P4 (10 µM) or DMSO (0.5%).

**Supplementary Figure 3: Mean activation of steroids is correlated with P4-inhibition.** Correlation plot between mean activation and mean P4-inhibition of of 72 steroids (42 validated steroids from the Prestwick library and 30 additional steroids generated from pharmacophore and statistical regression models). Each dot represents the mean maximal amplitude (%) triggered by individual steroids (mean activation) or in the presence of P4 (mean inhibition). R^2^ = 0.8808


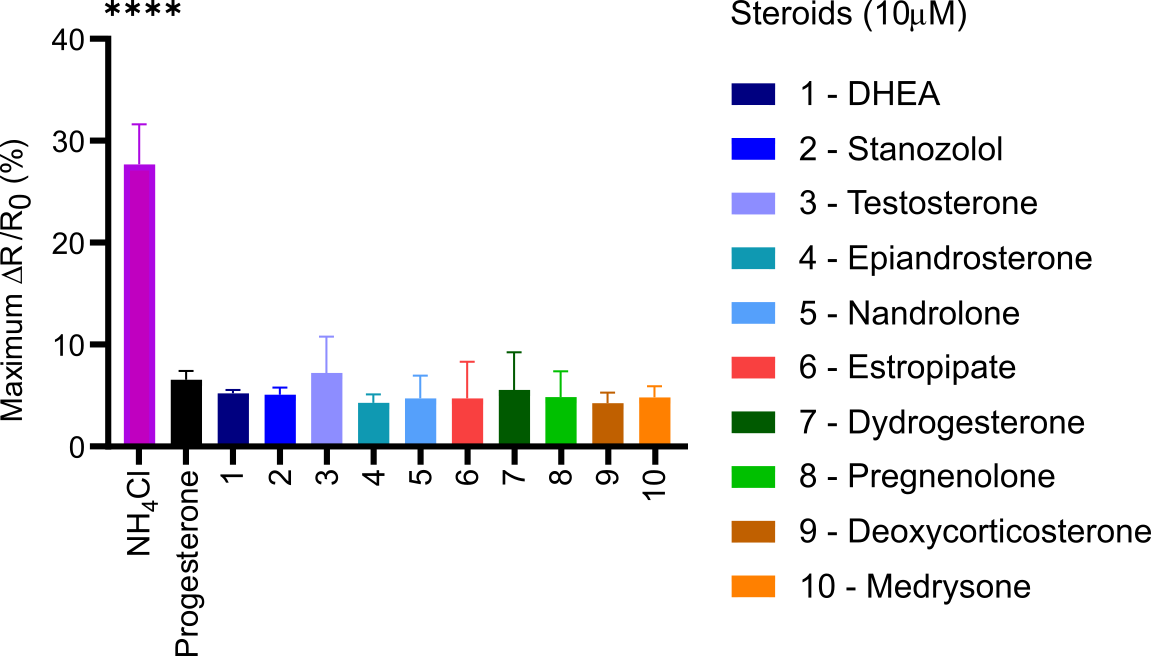


**Supplementary Figure 4: Steroids do not induce an increase in intracellular pH.** Changes in intracellular pH (pH_i_) were measured in sperm loaded with the pH-indicator BCECF-AM (2.5 µM) for 30 minutes at 37°C. Differences in pHi, reflected by changes in the fluorescence ratio, were analyzed as ∆R/R_0_ (%) and were monitored before and after the injection of steroids (10 µM). DMSO 0.5% was used as a vehicle control and NH_4_Cl (30mM) as a positive control. Data are presented as the mean of three independent experiments with error bars representing the standard deviation (SD); p (****)<0.0001.


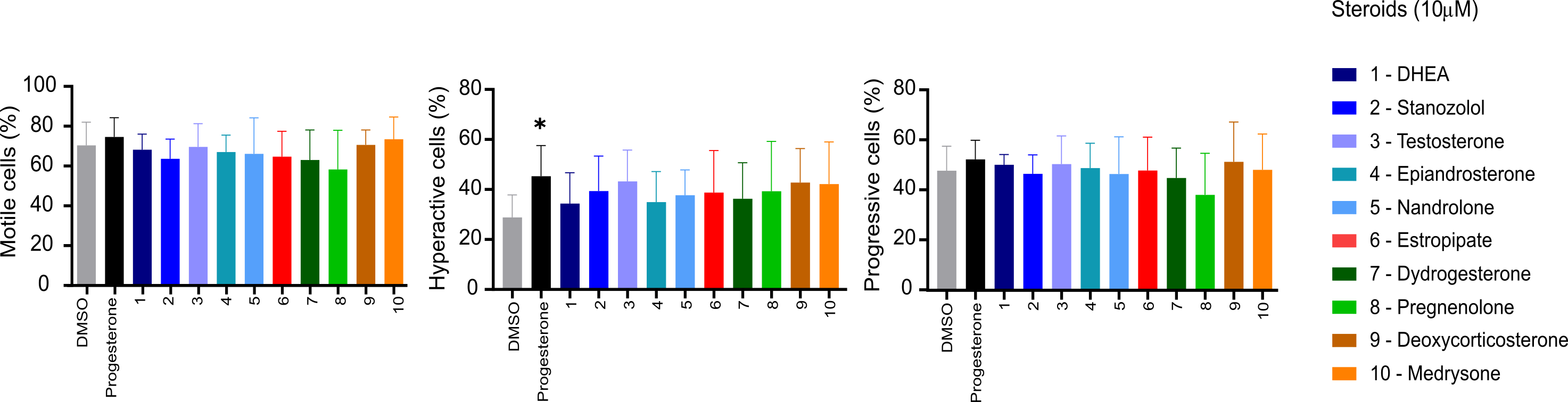


**A**

**B**

**C**

**Supplementary Figure 5: Effect of steroids on sperm motility *in vitro*.** Sperm motility was assessed using a computer-assisted sperm analysis (CASA, Hamilton Throne Ceros II, UK). Swim-up prepared sperm cells were diluted to a concentration of 12.5 x 10^6^/mL in HTF+ and were allowed to capacitate for 3 hours at 37°C in HTF+. Capacitated sperm were then incubated for an additional hour in the absence (DMSO) or presence of steroids, progesterone, or PGE1 at 10µM. A 2 µL aliquot was placed in a counting chamber with a 20 µm depth (Leja, Nieuw-Vennep, The Netherlands), and a minimum of 200 spermatozoa in at least five randomly selected fields were captured. Progressive motility was classified based on the average path velocity (VAP) with the following parameters: slow ≤1 > medium ≤ 25 > rapid. Hyperactive cells were classified based on the following parameters: VAP > 50 µm/s, a curvilinear velocity (VCL) > 90 µm/s, and an amplitude of lateral head (ALH) > 5 µm. None of the ten selected steroids induced a significant change in (**A**) total motility, (**B**) hyperactive motility, or (**C**) progressive motility. All data were compared with vehicle control DMSO. p (*)<0.0, 5) and are shown as the mean of n>4 independent experiments with error bars representing the standard deviation (SD).


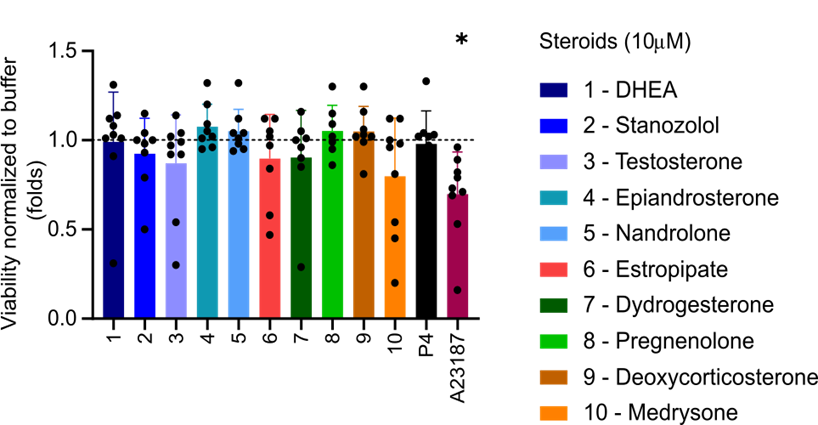


**Supplementary Figure 6: Viability of sperm cells in the presence of steroids.** Cell viability was assessed by flow cytometry of cells incubated with propidium iodide (PI). Cells were incubated with steroids (10 µM) for 1 hour or positive control ionophore A23187 (2 µM) for 45 minutes. Human sperm viability is not altered in the presence of the steroids at 10 µM. Data are presented as the mean of 9 independent experiments with error bars representing the standard deviation (SD); p (*)<0.05.

**Supplementary Table 1: Summary list of the 42 validated steroids and the 30 additional predicted steroids by SAR in order of increasing potency.** Summary table of all 42 validated steroids out of the 90 steroids from the Prestwick library (highlighted blue) and the additional 30 predicted steroids (highlighted orange). EC_50_s and IC_50_s are in µM; results are pooled from 3 independent experiments.

| **Chemical name** | **Max amplitude (%) (± SD)** | **Max P4 inhibition (%)**  **(± SD)** | **Physiological presence** | **Mean EC_50_ µM (±SD)** | **Mean IC_50_ (P4) µM (±SD)** | **Mean IC_50_ (PGE1)  µM (±SD)** |
| --- | --- | --- | --- | --- | --- | --- |
| progesterone | **100 (±0)** | **79 (±7)** | Yes | **0.002 (±0.001)** | **0.006 (±0.002)** | **NA** |
| S65 | **91 (±9)** | **95 (±1)** | No | **0.032 (±0.009)** | **0.046 (±0.008)** | **ND** |
| pregnenolone | **73 (±5)** | **82 (±5)** | Yes | **0.070 (±0.016)** | **0.199 (±0.008)** | **0.011**  **(±0.005)** |
| S46 | **141 (±32)** | **90 (±6)** | No | **0.073 (±0.02)** | **0.138 (±0.03)** | **NA** |
| stanozolol | **79 (±7)** | **96 (±2)** | No | **0.081 (±0.023)** | **0.190 (±0.045)** | **0.117**  **(±0.046)** |
| dydrogesterone | **84 (±12)** | **94 (±4)** | No | **0.104 (±0.103)** | **0.240 (±0.042)** | **44.84**  **(±25.54)** |
| S67 | **80 (±3)** | **86 (±3)** | No | **0.108 (±0.03)** | **0.253 (±0.067)** | **ND** |
| estropipate | **106 (±7)** | **90 (±10)** | No | **0.159 (±0.373)** | **0.280 (±0.01)** | **0.282**  **(±0.373)** |
| epiandrosterone | **78 (±5)** | **72 (±2)** | Yes | **0.207 (±0.033)** | **0.625 (±0.102)** | **NA** |
| medrysone | **104 (±17)** | **83 (±4)** | No | **0.230 (±0.103)** | **0.471 (±0.275)** | **NA** |
| S85 | **99 (±6)** | **96 (±2)** | No | **0.259 (±0.042)** | **0.495 (±0.051)** | **ND** |
| S64 | **65 (±6)** | **55 (±1)** | No | **0.265 (±0.079)** | **0.682 (±0.068)** | **ND** |
| estrone | **49 (±7)** | **67 (±2)** | Yes | **0.267 (±0.016)** | **0.560 (±0.051)** | **NA** |
| S76 | **57 (±6)** | **52 (±3)** | No | **0.277 (±0.006)** | **1.441 (±0.835)** | **ND** |
| S61 | **102 (±6)** | **97 (±1)** | No | **0.28 (±0.68)** | **0.25 (±0.048)** | **ND** |
| deoxycorticosterone | **94 (±12)** | **86 (±3)** | Yes | **0.295 (±0.062)** | **0.523 (±0.081)** | **NA** |
| nandrolone | **98 (±9)** | **85 (±1)** | No | **0.306 (±0.033)** | **0.729 (±0.109)** | **NA** |
| S62 | **82 (±7)** | **82 (±6)** | Yes | **0.33 (±0.078)** | **0.802 (±0.142)** | **ND** |
| formestane | **71 (±4)** | **70 (±8)** | No | **0.353 (±0.088)** | **1.216 (±0.442)** | **NA** |
| S75 | **37 (±3)** | **45 (±6)** | No | **0.358 (±0.182)** | **0.954 (±0.31)** | **ND** |
| S84 | **103 (±8)** | **97 (±2)** | No | **0.39 (±0.108)** | **0.198 (±0.049)** | **ND** |
| testosterone | **87 (±6)** | **81 (±6)** | Yes | **0.407 (±0.062)** | **1.092 (±0.157)** | **NA** |
| S45 | **125 (±18)** | **86 (±10)** | No | **0.445 (±0.098)** | **0.568 (±0.14)** | **ND** |
| exemestane | **63 (±6)** | **56 (±2)** | No | **0.557 (±0.12)** | **0.309 (±1.113)** | **NA** |
| dehydroisoandosterone 3-acetate | **77 (±3)** | **82 (±6)** | Yes | **0.584 (±0.043)** | **1.692 (±0.488)** | **3.704**  **(±0.961)** |
| fluorometholone | **42 (±1)** | **29 (±12)** | No | **0.702 (±0.329)** | **6.505 (±11.59)** | **NA** |
| estradiol-17 beta | **75 (±8)** | **67 (±3)** | Yes | **0.761 (±0.235)** | **1.451 (±0.225)** | **NA** |
| finasteride | **39 (±5)** | **18 (±6)** | No | **0.780 (±0.348)** | **NA** | **>100** |
| lithocholic acid | **59 (±4)** | **32 (±10)** | Yes | **0.909 (±0.079)** | **0.434 (±0.084)** | **NA** |
| 3-alpha-hydroxy-5-beta-androstan-17-one | **66 (±2)** | **66 (±2)** | No | **0.915 (±0.312)** | **1.517 (±0.216)** | **NA** |
| norgestimate | **79 (±3)** | **53 (±5)** | No | **0.918 (±1.111)** | **8.048 (±1.306)** | **NA** |
| equilin | **62 (±6)** | **50 (±11)** | No | **0.999 (±0.097)** | **1.85 (±0.416)** | **NA** |
| androsterone | **70 (±2)** | **61 (±6)** | Yes | **1.025 (±0.061)** | **2.108 (±0.759)** | **5.383**  **(±24.22)** |
| oxymetholone | **82 (±12)** | **62 (±9)** | No | **1.299 (±0.423)** | **4.738 (±1.64)** | **11.75**  **(±3.373)** |
| ethinylestradiol | **69 (±12)** | **50 (±14)** | No | **1.799 (±1.018)** | **3.363 (±1.02)** | **6.678**  **(±3.62)** |
| S82 | **60 (±10)** | **61 (±3)** | No | **1.906 (±0.026)** | **5.216 (±0.671)** | **ND** |
| S77 | **100 (±6)** | **80 (±2)** | No | **1.969 (±0.393)** | **3.073 (±0.149)** | **ND** |
| S44 | **102 (±18)** | **91 (±6)** | No | **2.132 (±1.311)** | **1.56 (±0.403)** | **ND** |
| S43 | **83 (±5)** | **77 (±3)** | Yes | **2.213 (±0.966)** | **3.122 (±0.884)** | **ND** |
| fulvestrant | **28 (±4)** | **22 (±6)** | No | **2.242 (±2.254)** | **6.953 (±2.474)** | **NA** |
| corticosterone | **55 (±0)** | **49 (±5)** | Yes | **2.936 (±0.486)** | **6.096 (±1.884)** | **NA** |
| S81 | **33 (±3)** | **35 (±5)** | No | **5.432 (±0.611)** | **12.9 (±15.5)** | **ND** |
| S78 | **38 (±19)** | **26 (±14)** | No | **58.07 (±208.7)** | **8.567 (±6.16)** | **ND** |
| norethindrone | **28 (±12)** | **22 (±5)** | No | **6.053 (±3.101)** | **4.346 (±3.028)** | **NA** |
| S71 | **56 (±10)** | **63 (±6)** | No | **7.023 (±9.242)** | **1.291 (±0.166)** | **ND** |
| canrenone | **69 (±22)** | **49 (±1)** | No | **7.506 (±6.101)** | **NA** | **NA** |
| urosiol | **25 (±8)** | **13 (±3)** | Yes | **7.888 (±2.169)** | **NA** | **12.17**  **(±6.159)** |
| mifepristone | **9 (±4)** | **25 (±25)** | No | **13.46 (±4.426)** | **10.95 (±5.198)** | **NA** |
| adrenosterone | **26 (±2)** | **24 (±3)** | No | **14.46 (±13.58)** | **30.75 (±342.6)** | **NA** |
| S68 | **37 (±14)** | **15 (±2)** | No | **15.34 (±18.21)** | **NA** | **ND** |
| lynestrenol | **59 (±24)** | **47 (±27)** | No | **17.26 (±5.523)** | **9.360 (±3.302)** | **11.71**  **(±3.784)** |
| ethynodiol diacetate | **35 (±14)** | **22 (±14)** | No | **17.68 (±5.114)** | **13.95 (±7.309)** | **12.19**  **(±9.644)** |
| S79 | **35 (±19)** | **13 (±4)** | No | **22.26 (±9.914)** | **NA** | **ND** |
| nomegestrol acetate | **19 (±12)** | **12 (±17)** | No | **22.65 (±7.749)** | **41.17 (±3.135)** | **51.99**  **(±24.92)** |
| canrenoic acid potassium salt | **34 (±2)** | **27 (±10)** | No | **23.35 (±28.88)** | **23.19 (±3.01)** | **16.34**  **(±9.27)** |
| chlormadinone acetate | **4 (±2)** | **0 (±15)** | No | **34.71 (±8.298)** | **NA** | **38.44**  **(±23.34)** |
| S73 | **16 (±2)** | **18 (±1)** | No | **76.54 (±80.74)** | **NA** | **ND** |
| budesonide | **7 (±2)** | **3 (±8)** | No | **NA** | **NA** | **>100** |
| ethisterone | **8 (±5)** | **10 (±9)** | No | **NA** | **5.128 (±60.47)** | **NA** |
| ethynylestradiol 3-methyl ether (mestranol) | **9 (±2)** | **4 (±3)** | No | **NA** | **NA** | **NA** |
| gestrinone | **2 (±2)** | **11 (±21)** | No | **NA** | **33.57 (±18.96)** | **NA** |
| megestrol acetate | **3 (±3)** | **-4 (±11)** | No | **NA** | **NA** | **NA** |
| norgestrel-(-)-d | **5 (±3)** | **3 (±5)** | No | **NA** | **75.56 (±2.899)** | **NA** |
| S80 | **10 (±1)** | **8 (±2)** | No | **NA** | **NA** | **ND** |
| S72 | **94 (±6)** | **93 (±3)** | No | **NA** | **NA** | **ND** |
| S66 | **31 (±18)** | **11 (±10)** | No | **NA** | **NA** | **ND** |
| S70 | **25 (±25)** | **8 (±7)** | No | **NA** | **NA** | **ND** |
| S69 | **19 (±17)** | **10 (±4)** | No | **NA** | **NA** | **ND** |
| S74 | **7 (±2)** | **6 (±8)** | No | **NA** | **NA** | **ND** |
| S83 | **3 (±1)** | **0 (±9)** | No | **NA** | **NA** | **ND** |
| S63 | **6 (±3)** | **4 (±3)** | No | **NA** | **NA** | **ND** |

**Supplementary Table 2.** **Estimated parameters and standard errors (SD) of fixed effects parameters of the beta zero-inflated semi-parametric model.** Variables whose interaction has been considered in the model are denoted with a vertical line. p (***) < 0.001, p (*) < 0.05, p (.) < 0.1, p (,) < 1.

|  | **Estimate** | **SD** | **t-value** | **p-value** |  |
| --- | --- | --- | --- | --- | --- |
| (Intercept) | 2.0206 | 0.5167 | 3.9104 | 0.0001 | *** |
| QPlogPo/w | 0.2000 | 0.1277 | 1.5660 | 0.1187 |  |
| QPlogHERG | 1.1337 | 0.2076 | 5.4598 | 1.2E-07 | *** |
| number of amide groups \| dip^2/V | 42.6134 | 16.7341 | 2.5465 | 0.0115 | * |
| QplogPo/w \| dip^2/V | 6.5199 | 2.7610 | 2.3614 | 0.0190 | * |
| y-component of dipole moment \| dip^2/V | 3.4160 | 1.6682 | 2.0477 | 0.0417 | * |
| number of rotatable bonds \| number of h-bond donors \| dip^2/V | 1.4152 | 0.7905 | 1.7902 | 0.0747 | , |
| number of h-bond donors \| QPlogPo/w \| dip^2/V | -3.5029 | 2.0682 | -1.6937 | 0.0916 | , |
| number of h-bond donors \| x-component of dipole moment \| dip^2/V | 1.3395 | 0.5290 | 2.5325 | 0.0120 | * |
| QPlogPo/w \| z-component of dipole moment \| dip^2/V | 0.8904 | 0.3476 | 2.5615 | 0.0110 | * |
| y-component of dipole moment \| z-component of dipole moment \| dip^2/V | 0.9267 | 0.4408 | 2.1024 | 0.0366 | * |

**References:**

DIXON, S., SMONDYREV, A. & RAO, S. 2006. PHASE: A Novel Approach to Pharmacophore Modeling and 3D Database Searching. *Chemical biology & drug design,* 67**,** 370-2.

IMRIE, F., HADFIELD, T. E., BRADLEY, A. R. & DEANE, C. M. 2021. Deep generative design with 3D pharmacophoric constraints. *Chemical Science,* 12**,** 14577-14589.

OSPINA, R. & FERRARI, S. L. P. 2012. A general class of zero-or-one inflated beta regression models. *Computational Statistics & Data Analysis,* 56**,** 1609-1623.

AKAIKE, H. (ed.) 1973. *Information theory and an extension of the maximum likelihood principle,*Budapest, Hungary: Akadémiai Kiadó.

STASINOPOULOS, M. D., RIGBY, R. A., HELLER, G. Z., VOUDOURIS, V. & DE BASTIANI, F. 2017. *Flexible regression and smoothing: using GAMLSS in R*, CRC Press.

ZOU, H. 2006. The Adaptive Lasso and Its Oracle Properties. *Journal of the American Statistical Association,* 101**,** 1418-1429.
